# One Step Closer to a Molecular Level Understanding of the Tear Film Lipid Layer by Surface X‑ray Scattering

**DOI:** 10.1021/acs.langmuir.5c01920

**Published:** 2025-08-25

**Authors:** Ryan M. Trevorah, Henrik Stubb, Mira Viljanen, Henrik Mäkinen, Tuomo Viitaja, Julia Sevón, Oleg Konovalov, Maciej Jankowski, Arnaud Hemmerle, Filip S. Ekholm, Kirsi J. Svedström

**Affiliations:** † Department of Physics, 3835 University of Helsinki, P.O. Box 64, FI-00014 Helsinki, Finland; ‡ Department of Chemistry, University of Helsinki, P.O. Box 55, FI-00014 Helsinki, Finland; § Ophthalmology, University of Helsinki and Helsinki University Hospital, Haartmaninkatu 8, FI-00290 Helsinki, Finland; ∥ 55553 The European Synchrotron Radiation Facility—ESRF, 71 Avenue des Martyrs, CS 40220, 38043 Grenoble Cedex 9, France; ⊥ 55536 Synchrotron SOLEIL, L’Orme des Merisiers, Départementale 128, 91190 Saint-Aubin, France

## Abstract

The tear film lipid layer (TFLL) is the outermost layer of the tear film and forms a barrier between the eye and the environment. While the TFLL is important in maintaining ocular surface health, there remains a curtain of mystery surrounding its structure and function on the molecular level. This is the result of the complex composition of the lipid film, the challenging dynamic environment in which it is present and missing molecular level information on the properties displayed by its lipid constituents. We recently assessed whether state-of-the-art surface X-ray scattering techniques can be employed to study the properties of films formed by individual tear film lipids and found this approach to bear significant potential in addressing the current unknown parameters of these substrates. Herein, we perform a follow-up study utilizing an expanded library of molecules in order to uncover general trends displayed by distinct tear film lipid classes. Through the use of grazing incidence X-ray diffraction and X-ray reflectivity techniques, we determine the lattice distances, molecular tilt angles and film thickness of representative lipids featuring variations in branching patterns and chain lengths and take an important step toward a deeper understanding of the molecular level structure and function of individual tear film lipids.

## Graphical abstract

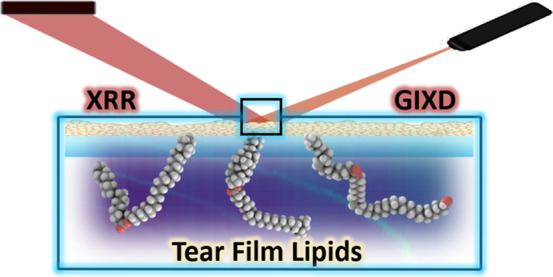

## Introduction

The tear film lipid layer (TFLL) forms the outermost protective layer of the tear film and plays a central role in the upkeep of ocular surface health and vision. The TFLL is present in a challenging environment which has led to the evolution of lipid species with specific molecular structures and properties. The composition of the TFLL is complex. In fact, up to 600 distinct lipid species have been reported in the natural biofilm which is secreted by the meibomian glands.^1^ While lipidomic studies have been able to shed light on the structure of individual components and proportions of lipid species and classes in the TFLL,^2–8^ there are still many unknown factors related to the fundamental physical properties and potential roles of individual lipids and how they collaborate to provide and sustain TFLL structure and function.^9^ Addressing these questions on a molecular level requires in-depth insights on the properties displayed by both individual tear film lipids and their more complex compositions.

Recently, we assessed whether advanced synchrotron surface scattering methods such as grazing incidence X-ray diffraction (GIXD) and X-ray reflectivity (XRR) can be employed to increase our knowledge on the fundamental properties displayed by tear film lipid films.^10^ We found these techniques to be promising for studying the behavior of tear film lipids at the aqueous–air interface under simulated physiological conditions and noted several similarities to the properties reported previously for meibum.^11^ In more detail, we studied the properties of four representative tear film lipids, one from each of the following categories: *O*-acyl-ω-hydroxy fatty acid (OAHFA), type II diester (type II DiE), wax ester (WE) and cholesteryl ester (CE) (Figure 1). These lipid classes represent key contributors to the polar and nonpolar part of the TFLL and have been proposed to play crucial roles in several suggested models of TFLL function.^9,12–14^

**Figure 1:**
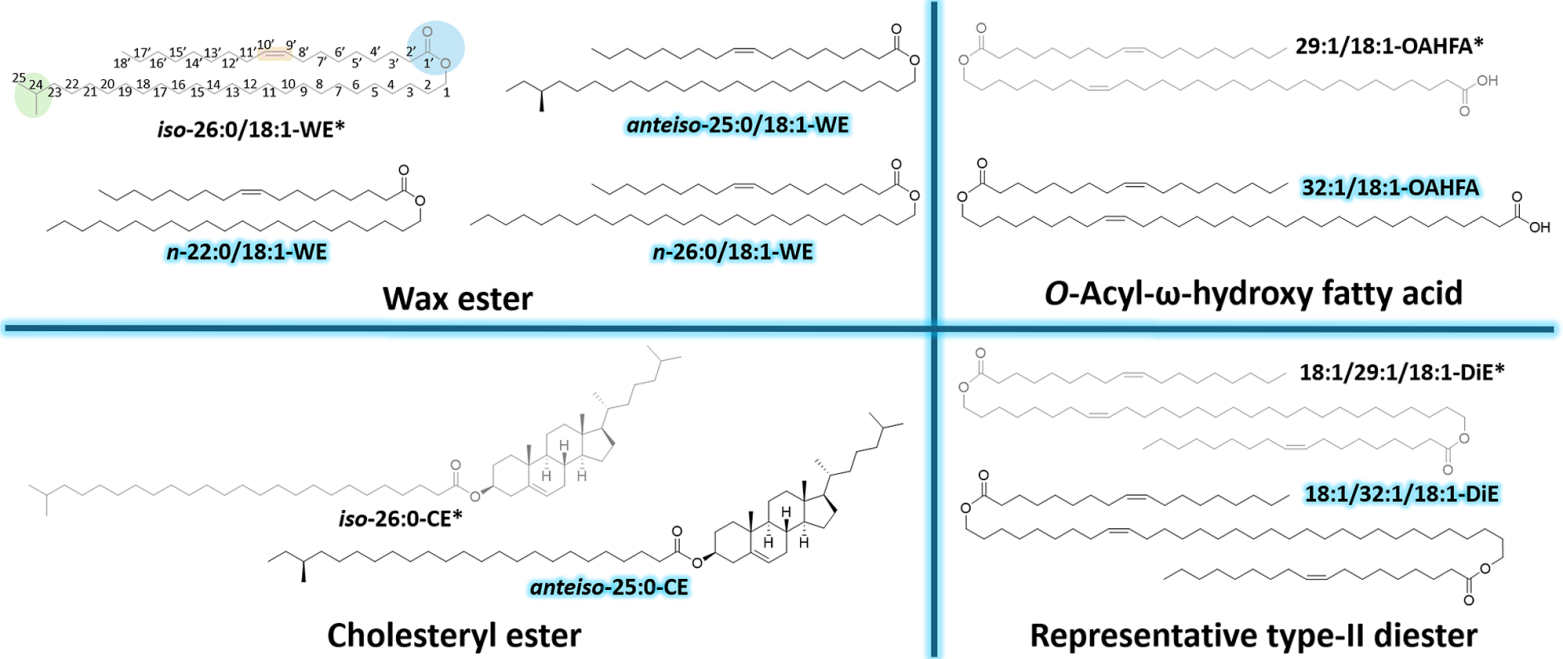
Molecular structures of tear film lipids studied by surface X-ray scattering techniques. The gray structures/ones marked with an * were studied previously^10^ and the structures which appear in black/names highlighted are studied here for the first time. Please see Table S4 for additional insights on the origin of these lipid species. One example of the correlation between the molecular structure and naming abbreviations utilized is shown in the top left corner for *iso*-26:0/18:1-WE. *Iso* refers to the branching pattern whereas the 26:0/18:1 refers to the number of carbon atoms and double bonds in the carbon chains and WE describes the tear film lipid class that the molecule belongs to.

In this follow-up study, the aim was to expand upon our earlier work in order to observe whether the results concerning crystalline lattice types, lattice parameters, molecular tilt angles (angle to the surface normal) and film thickness represented general trends or whether there are differences in these which depend on structural factors such as branching and chain length. In this follow-up study, the most abundant species from the OAHFA and type II DiE classes and species with altered branching patterns from the WE and CE categories were included (Figure 1). Our results reveal that while branching and chain length do affect the fundamental physical profiles of tear film lipid species, there are also observable general trends which we believe will be essential to deciphering the molecular level structure and mechanisms behind TFLL function.

## Experimental Section

### Synchrotron Surface Scattering Studies

Synchrotron experiments were conducted by following the same protocols as used and detailed in our previous study.^10^ GIXD and XRR measurements were performed at beamline ID10 at the European Synchrotron Research Facility (ESRF) in Grenoble, France. The X-ray energy was 22.0 keV. For GIXD, a Dectris Mythen 2K detector was used. For XRR, a Maxipix detector (2560 × 256 pixels) was used. Further GIXD measurements were also collected at the SIRIUS beamline, SOLEIL Synchrotron in Saint-Aubin, France with an X-ray energy of 8.0 keV and a 2D Pilatus3 1M detector (Dectris, Switzerland) with a Soller collimator.^15^ In each of these experiments, attenuators were employed in the beam path as beam damage was observed when the sample was exposed to the full flux. The Langmuir trough experiments were performed within an enclosure and a continuous helium flow was utilized to minimize potential oxidation of the lipids. The lipid samples (5 mM chloroform solutions) were administered onto a subphase of a PBS buffer solution prior to the studies and the surface pressure was measured using a Wilhelmy plate. The chloroform was allowed to evaporate for 5 min after which the film was compressed, and the desired surface pressure was maintained for the duration of the experiment.

The GIXD data was analyzed based on the *NN* (nearest neighbor tilted) or *NNN* (next nearest neighbor tilted) phase reflections observed in the GIXD patterns and using Python code with Gaussian peak fitting on them and the same equations as given in the Supporting Information (see page 4) of our previous study.^10^

The linear compressibility (*X*) corresponds to the relative distortion in the chosen lattice direction for applied isotropic stress and was thus computed by using the following equation

$$X=-\frac{1}{u}\;\frac{du}{d\pi }$$

where *u* is the length (in chosen direction) and π is the surface pressure.

The XRR data was fitted with slab models (fitted parameters given in the Supporting Information in Tables S2 and S3) using the EasyReflectometry^16^ and Refl1d programs.

## Results and Discussion

### Selection of Substrates

The TFLL consists of approximately 40–70% CEs, 30–50% WEs, 3–4% OAHFAs,^17^ 2–8% DiEs^18^ and up to 10% or more of other lipids (acylglycerols, phospholipids, ceramides, etc.). Both in our recent study,^10^ and in this one, we focus on the properties displayed by WEs, CEs, OAHFAs and DiEs. Together, these lipid classes make crucial contributions to the polar and nonpolar parts of the lipid layer and their species represent ∼90% of the total TFLL composition. In our recent study, we focused on uncovering the properties of one polar lipid (TFLL context^19^), namely: (21*Z*)-29-oleoyloxynonacos-21-enoic acid (29:1/18:1-OAHFA), one semipolar lipid, namely: (8*Z*)-1,29-dioleoyloxynonacos-8-ene (18:1/29:1/18:1-type II DiE), and two nonpolar lipids, namely: cholesteryl 24-methylpentacosanoate (*iso*-26:0-CE) and 24-methylpentacosyl oleate (*iso*-26:0-WE). The 29:1/18:1-OAHFA and 18:1/29:1/18:1-type II DiE represented the average chain length species of this type in the TFLL, whereas the *iso*-26:0-CE and *iso*-26:0-WE represented the most abundant species in these lipid classes.

In this study, we decided to assess the properties of the most abundant polar TFLL OAHFA, the 32:1/18:1-OAHFA, and the structurally related and relatively abundant semipolar diester 18:1/32:1/18:1-type II DiE (Figure 1). These were considered relevant substrates because the 32:1/18:1-OAHFA makes up about 30% of the OAHFAs,^7^ and the 32:1-parent chain makes up over 50% of α,ω-diols found in meibum.^20^ These compounds are not commercially available, and thus in order to be able to study their properties we first had to device and complete a multistep total synthesis route.

The synthesis of 32:1/18:1-OAHFA has been reported previously^21,22^ on a few occasions whereas the synthesis of the structurally related type II DiE has not. In some of the previous studies, the NMR-data and spectra confirming the chemical structures of intermediate products and the final compound are missing whereas in others, the product appears as an undefined mixture of *E*/*Z*-isomers based on the NMR-spectra supplied. Thus, we considered it important to revisit the synthesis of the 32:1/18:1-OAHFA and complement the data existing in the literature while simultaneously providing a route to the 18:1/32:1/18 type II DiE. Altogether, the synthesis of the naturally occurring 32:1/18:1-OAHFA and related 18:1/32:1/18:1-type II DiE could both be performed over 10 steps with overall yields of 14% and 15%, respectively (Scheme 1). The end products, as well as all intermediates, were characterized in detail by high resolution mass spectrometry (HRMS) and NMR-spectroscopy whereas the melting points were also determined for the two naturally occurring tear film lipids: the 32:1/18:1-OAHFA and 18:1/32:1/18:1-type II DiE. A more detailed discussion on the synthesis and structural characterization part is provided together with the synthetic protocols, substrate specific analytical data and reference ^1^H/^13^C NMR spectra in the Supporting Information.

**Scheme 1:**
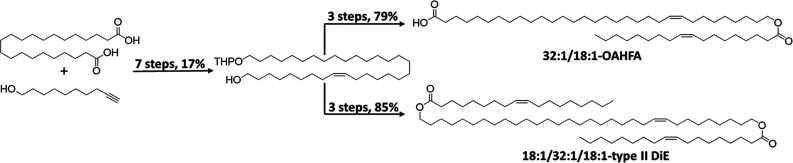
An Overview of the Synthesis Routes to 32:1/18:1-OAHFA and 18:1/32:1/18:1-Type II DiE

For the nonpolar species, we decided to expand upon our previous studies on *iso*-branched species by investigating CEs/WEs with other branching patterns. We considered this to be important since these are the most abundant tear film lipid classes and lipidomic studies have shown that the TFLL contains a mixture of straight-chain, *iso*-branched, and *anteiso*-branched WEs/CEs.^23^ The molecules selected for this study were (Figure 1): hexacosyl oleate (*n*-26:0/18:1-WE), docosyl oleate (*n*-22:0/18:1-WE), (22*S*)-22-methyltetracosanyl oleate (*anteiso*-25:0/18:1-WE) and cholesteryl (22′*S*)-22′-methyltetracosanoate (*anteiso*-25:0-CE). These were considered good substrates because they have similar chain lengths as the previously studied *iso*-branched species thus allowing an assessment of changes in surface properties induced by chain branching. The total synthesis and detailed structural characterization of these species have been reported by our team recently^24,25^ and will not be discussed in more detail herein.

### Synchrotron GIXD and XRR Studies on the New Set of Tear Film Lipids

With access to the extended tear film lipid library and insights on the fundamental biophysical profiles of individual lipids (see information in recent publications^10,25,26^ ), we shifted our focus to studying the properties of films formed by the new tear film lipid species with the following surface scattering techniques: GIXD and XRR. These techniques provide complementary data sets which yield insights into the horizontal (in-plane) and vertical dimensions of film structure. In more detail, we studied the films formed by the lipids at the aqueous interface under conditions mimicking those at the ocular surface by using an aqueous subphase of similar pH and electrolyte concentration as the environment at the ocular surface and performing the experiments at, or close to, ocular surface temperature (at 30 and 35 °C) and at selected surface pressures (between 5 and 40 mN/m) which depended on the stability of the lipid films. Through the use of GIXD, the goal was to obtain quantitative molecular lattice distances and tilt angles which are important for deducing the molecular architecture formed at the aqueous surface. Through XRR measurements, the goal was to analyze the thickness and electron density profile of the lipid films. The combined information from these experiments yields important insights on the intrinsic properties of individual tear film lipid classes and is an integral part of advancing the molecular level understanding of these interesting species and their potential contributions to the TFLL overall. The discussion below will consider comparisons between the GIXD and XRR results highlighting the properties of the distinct lipid classes, whereas our recent results,^10^ as well as those reported earlier for meibum,^11^ will be utilized as reference points.

A summary of the GIXD results is presented in Table 1 alongside the reference values from our previous study^10^ and those reported for meibum.^11^ While some general trends could be observed for individual lipid classes, deviations between lipid species were likewise noted. We started by assessing the film behavior of the polar 32:1/18:1-OAHFA which would be expected to reside in the polar lipid layer in direct contact with the aqueous interface.^9^ At physiological conditions (*T* = 35 °C, *P* = 30 mN/m), the GIXD pattern of 32:1/18:1-OAHFA showed a pair of peaks thereby indicating an *NN* phase in a similar fashion as that observed previously for the 29:1/18:1-OAHFA (presenting the average length tear film OAHFA). In addition, the lattice parameters for these two species were strikingly similar. Nevertheless, differences could also be observed. For example, the GIXD pattern of 32:1/18:1-OAHFA was more complex, and several scattering peaks were observed in addition to the two *NN* identified reflections (Figure 2). This indicates the possible coexistence of multiple phases, a finding that is supported by Brewster angle microscopy (BAM) imaging of the film structure as well (see Figure S28). This could also be linked to the observed deviations for the in-plane coherence lengths (i.e. the average size of the mosaic type crystalline regions), which were notably smaller (45–330 Å) for 32:1/18:1-OAHFA when compared to the values of 29:1/18:1-OAHFA (200–460 Å).

**Table 1:**
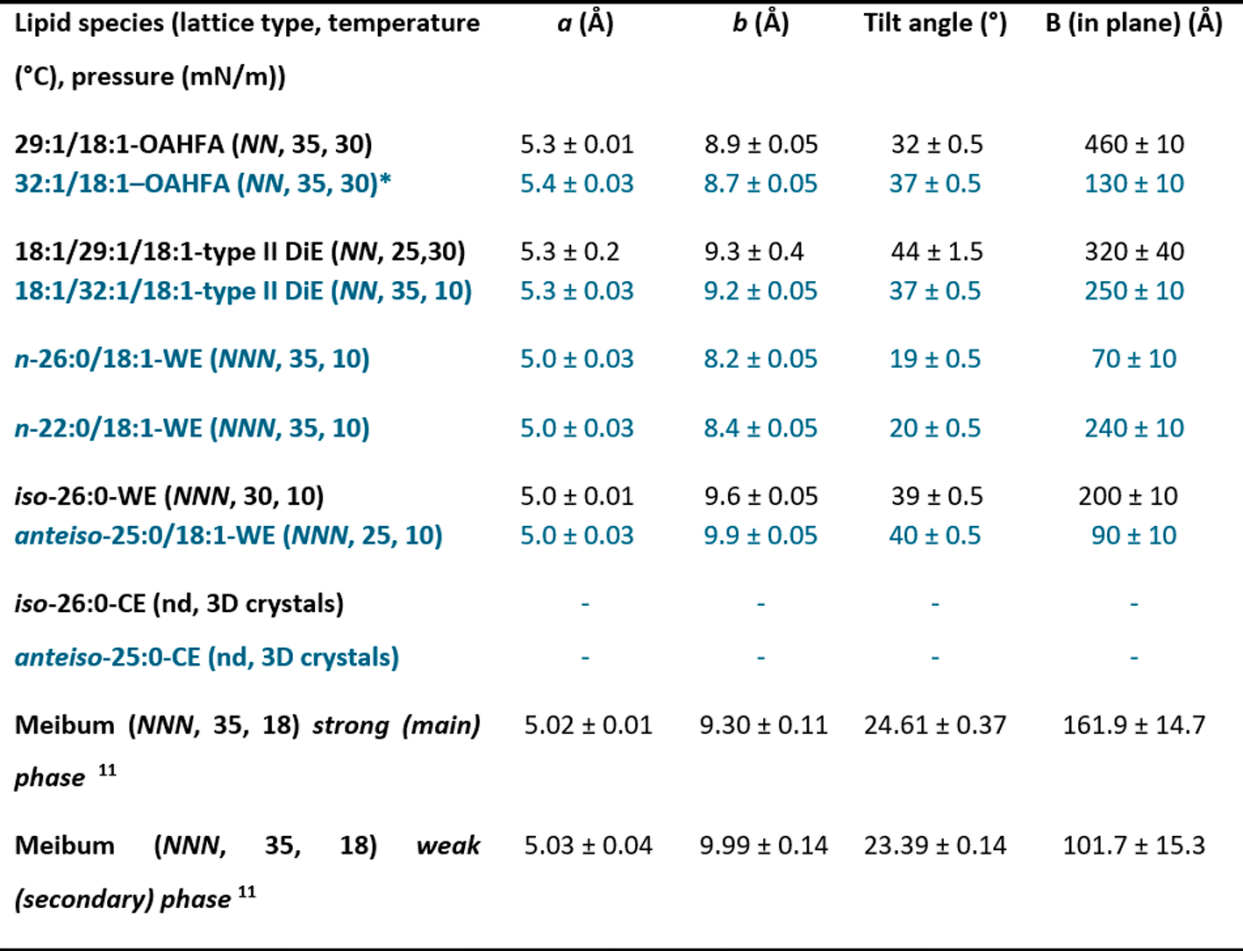
A Summary of the GIXD Results: Lattice Parameters (*a* and *b*), Molecular Tilt Angle and the Average In-Plane Coherence Length (*B*)­^a^

a For comparison, the literature values for human meibum determined by Leiske et al.^11^ and our previous results on tear film lipids^10^ are shown. In the table, the new lipid species studied and results supplied are marked with a blue color. Moreover, this table presents the values determined at the most physiologically relevant measurement point, please see the Table S1 for the additional results. *NN* = nearest neighbor tilted phase. *NNN =* next nearest neighbor tilted phase. nd = not detected. * = multiphase.

**Figure 2:**
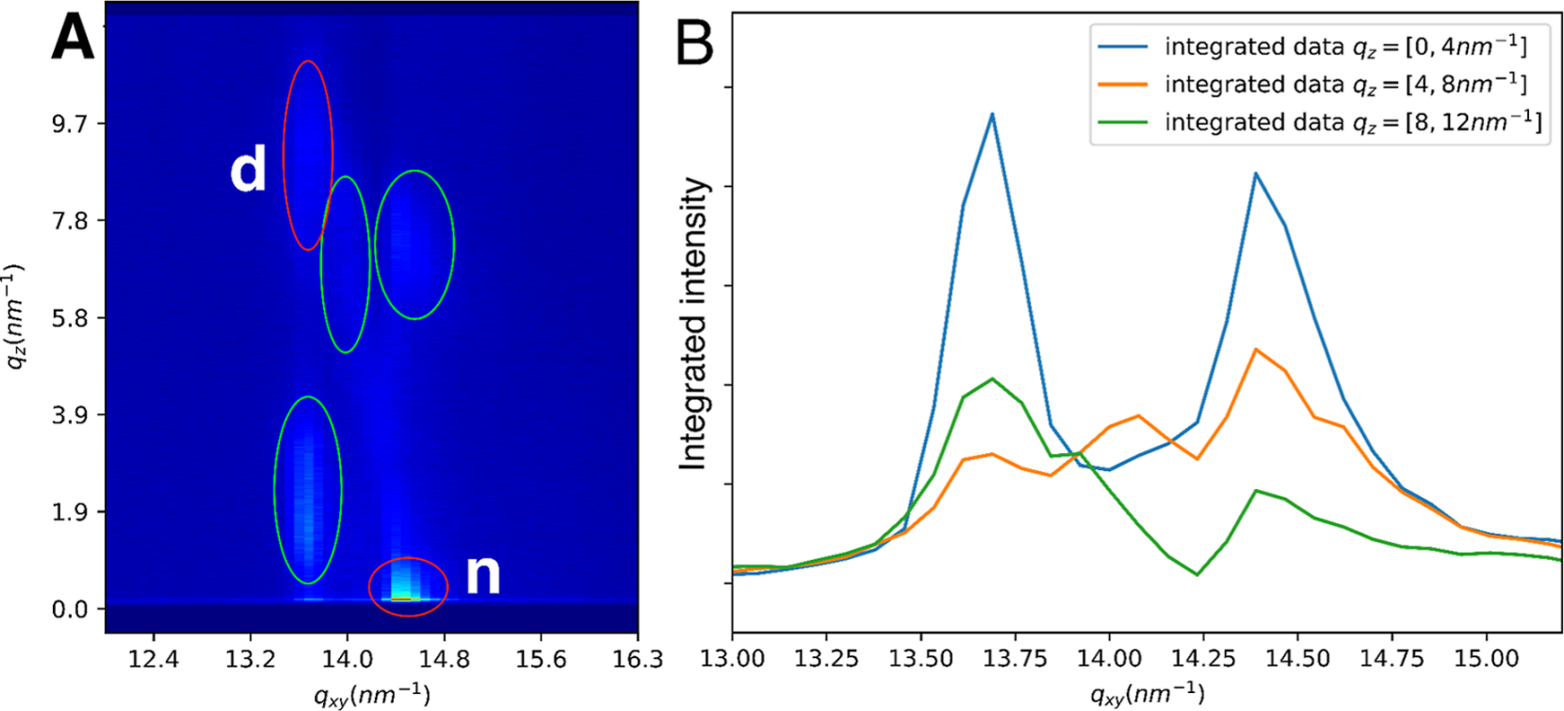
(A) 32:1/18:1-OAHFA GIXD pattern at 35 °C, 30 mN/m, showing the main (*NN* tilted) phase (marked with red ovals) besides other reflections (marked with green ovals); (B) integrated intensities of 32:1/18:1-OAHFA. Note: d = degenerate peak (*11* and *1-1*); n = nondegenerate peak (*02*).

In addition, the surface pressure dependent molecular tilt angle changes were found to follow distinct trends for the 29:1/18:1-OAHFA and the 32:1/18:1-OAHFA. For the 32:1/18:1-OAHFA, the molecular tilt angle was the same or increased as a function of increased surface pressure which is in line with the findings on the other studied tear film lipids thus far (see Table S1), with the exception of 29:1/18:1-OAHFA. It is not yet clear whether this behavior is affected by overall chain length or whether the deviating trends are connected to even/odd numbered parent chains in the OAHFA-species. Investigating this relationship in more detail would require access to a larger series of tear film OAHFAs and non-natural structural analogues, which is a topic that we may return to as part of future work.

We proceeded by studying the molecular level compressibility of these films, another dynamic feature which is closely related to the function of the TFLL. In our previous study,^10^ we found that for 29:1/18:1-OAHFA, the lattice distance was decreasing as a function of increasing surface pressure. Based on the GIXD patterns of 29:1/18:1-OAHFA,^10^ the linear compressibility values were (at 25 °C, 30 °C and 35 °C) on the *a*-axis: 0.8 ± 0.2 m/N, and, on the *b*-axis: 0.3 ± 0.1 m/N (negligible at 35 °C). For the most abundant tear film OAHFA, 32:1/18:1-OAHFA, the compressibility values were relatively similar (at 30 °C: negligible in the *a*-axis direction, 0.5 ± 0.2 m/N in the *b*-axis direction; at 35 °C: negligible both in *a*- and *b*-axis direction). These values indicate that the films are relatively rigid, and that the rigidity is possibly increased as a function of increased chain length. Moreover, we find it plausible that our studies on the average length/most abundant tear film OAHFAs have revealed in which range the optimum spot might be when it comes to their contributions to the rigidity of the polar lipid film. While previous studies on tear film OAHFAs have not been performed, the values obtained here correspond to the lower end of the linear compressibility values reported for behenic acid.^27^ On a more general level, low values such as these, have also been noted for solid untilted phases and crystalline polymers.^27,28^

The XRR results of the 32:1/18:1-OAHFA were also interesting to compare to those obtained previously for the 29:1/18:1-OAHFA. The 29:1/18:1-OAHFA showed clear oscillatory XRR curves which were considered to correspond to a monolayer with two electron densities (one for the polar headgroup and one for the hydrophobic tail) and a total layer thickness of 54 Å. Interestingly, the 32:1/18:1-OAHFA showed a more complex XRR pattern (especially above 20 mN/m and at 35 °C, Figure 3A): the expected oscillatory features of a monolayer could be observed (clearest at 10–20 mN/m at 30 °C), but on top of this, another (higher frequency) pattern was present which we interpreted as the formation of Bragg peaks. These findings might suggest that the 32:1/18:1-OAHFA film consists of two distinct crystalline regions, i.e. regions composed of a pure monolayer structure and regions in which additional lamellar multilayer structures form.

**Figure 3:**
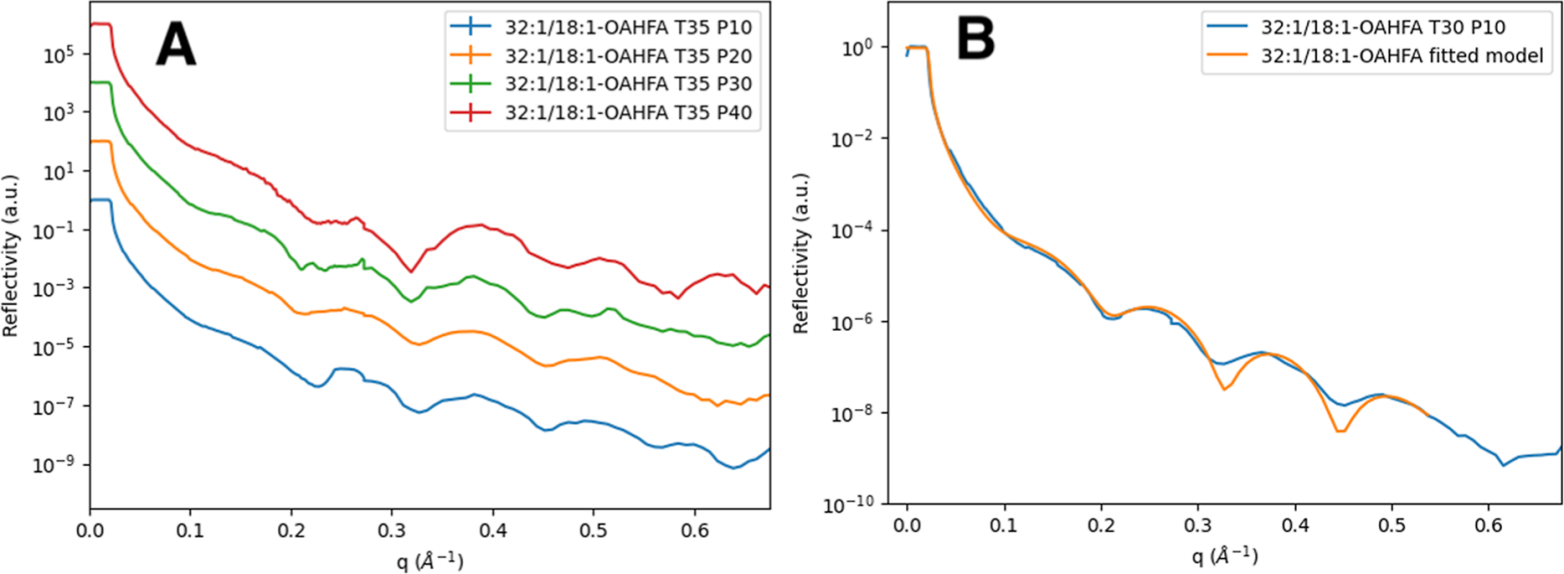
(A) XRR curves of 32:1/18:1-OAHFA (*T* = 35 °C) at various surface pressures (at 10 mN/m in blue, 20 mN/m in orange, 30 mN/m in green, 40 mN/m in red). The spectra have been created by combining two XRR scans (at *q* ∼ 0.27 Å^–1^) which cover the lower and higher *q* ranges, respectively. (B) Fitted monolayer-model into the experimental XRR data of 32:1/18:1-OAHFA at 30 °C and 10 mN/m (fitted parameter values given in Table S2).

Combined with the GIXD results on two possible crystalline phases, it could be hypothesized, that the regions organized as monolayers are connected to the *NN* phase, and the lamellar regions with the other crystalline phase. These observations on polymorphism and simultaneous existence of various crystalline regions are also supported by the findings obtained from BAM-imaging of the film structure (see Figure S28). Based on the fit for the XRR curve measured at 10 mN/m, the thickness of the monolayer region of the 32:1/18:1-OAHFA was 58 ± 1 Å indicating that a slight increase in chain length (+3 carbon atoms compared to 29:1/18:1-OAHFA) is accompanied by a slight increase in layer thickness (+4 Å compared to 29:1/18:1-OAHFA). The potential Bragg peaks were faint (see Figure 3A), but possible signs of them could be observed around 0.26 and 0.52 1/Å at 30 °C, and 0.26, 0.39, 0.52 1/Å at 35 °C in the surface pressure range of 20–30 mN/m, i.e. under physiological pressures and temperature. Interestingly, these positions would correspond to lamellar spacings (47 ± 1 Å) in the same range as those observed for WEs. Coincidence or not, it does seem like there are previously unrecognized similarities in the structural properties observed for distinct tear film lipid classes.

Next, we shifted our focus to the semipolar 18:1/32:1/18:1-type II DiE. There is not a great deal of insight available on the preferential location and specific roles of these species within the TFLL, i.e. it is not yet known if they reside in the polar or nonpolar lipid layer, or if they aid in creating a polarity gradient within the TFLL or in structuring intersections, or whether they play an important role in the dynamic behavior of the film. Thus, all possible structural information obtained by GIXD/XRR could increase the understanding of the roles of type II DiEs in the TFLL. The first thing to note is that the film formed by 18:1/32:1/18:1-type II DiE could not be studied at ocular surface pressures due to collapse (see surface pressure isotherm in Figure S28). Thus, it is likely that the contribution of these species to the TFLL structure and function is achieved through interactions with other lipid classes/species. Nevertheless, based on the GIXD data obtained, at ocular surface temperature, the 18:1/32:1/18:1-type II DiE (Figure S1) and the previously studied 18:1/29:1/18:1-type II DiE behaved in a similar manner. Both displayed an *NN* lattice type with similar lattice parameters and the in-plane coherence lengths were found to be in the same range (250 Å vs 320 Å). Elongation of the parent chain length was accompanied by a shortening of the in-plane coherence length in a similar fashion as noted for the OAHFA species above. Moreover, the lattice parameters are similar to the ones of the OAHFAs at ocular surface temperature and pressure (25–35 mN/m) and those reported for the main phase of meibum. This provides some insights into a framework through which meibum could generate its structure, however, to complete the picture it would be important to study the effects of interactions between distinct lipid classes and potentially other biomolecules present at the ocular surface as well.

The XRR spectra were also determined for 18:1/32:1/18:1-type II DiE. The XRR pattern for the type II DiE was more complex than for any of the other species studied, which was interpreted either to be due to the coexistence of multiple distinct phases or a multilayer structure with more alterations than just purely repeating monolayers (see Figure S4). An example of a multilayered model (a 7-slab model consisting of 3 repeating thicker layers (17–25 Å), which could correspond to carbon chain tails, with thinner (border/interval) layers (3–8 Å) possibly corresponding to the ester functional group) fitted into the XRR data and provided a rough view of how the layer thicknesses and electron densities could be distributed (see Table S3). While we were unable to fully generate the XRR patterns with a complete model, the complex behavior may in part suggest adaptive roles of DiEs within the TFLL. The multilayered structure (also suggested by the surface pressure isotherm shown in Figure S28) along with their semipolar nature indicates that these species could be important in structuring of intersections in the TFLL and/or forming a polarity gradient as the TFLL extends from the aqueous interface toward the air. This is in line with some of the current models suggested for TFLL structure and function.

Having studied the polar and semipolar lipid classes, we shifted our attention to the main constituents of the TFLL, i.e. the WE and CE species thought to reside mainly in the nonpolar lipid layer. There is a limited amount of surface X-ray scattering data on WEs overall, not to mention the species present in the TFLL. In our recent work,^10^ we studied the most abundant *iso*-26:0/18:1-WE at 30 °C and 10 mN/m and showed that it had an *NNN* lattice type with similar lattice parameters as those observed for meibum (and the other lipid species discussed above). Reminiscent of the type II DiE case, the studies on WEs could not be performed at ocular surface pressure and temperature because the films collapse prior to reaching these conditions as shown in our recent studies.^24,25^ Therefore, the investigations on the new set of WE species (*n*-26:0/18:1-WE, *n*-22:0/18:1-WE and the *anteiso*-25:0/18:1-WE) had to be performed at lower surface pressures. The closest values to the physiological state we could reach for each of the WEs are those given in Table 1. Nevertheless, we were interested in studying potential similarities and differences between species with distinct branching patterns as the considerable portion of WEs in the TFLL are branched. Under the applied conditions, the included WEs all existed in the *NNN* phase (Figures 4A, S2, and S3), and the lattice parameters were analogous to the ones observed earlier for *iso*-26:0/18:1-WE. A similar trend concerning in-plane coherence lengths was observed for the other species, i.e. the coherence lengths were shortened when the chain length was increased. In addition, *iso*- and *anteiso*-branching did influence the behavior of these molecules as the molecular tilt angles were found to be significantly larger than for the straight-chain species (∼2× larger). This put the tilt angles for the *iso*- and *anteiso*-branched species in the same range as the tilt angles observed for the most abundant OAHFA and type II DiE studied. It is unclear whether similarities between molecular tilt angles is a driving factor behind the formation of integrated structures with interesting properties, but it may very well be an important factor to consider when addressing the molecular level structure of tear film lipid compositions and the TFLL in the future.

**Figure 4:**
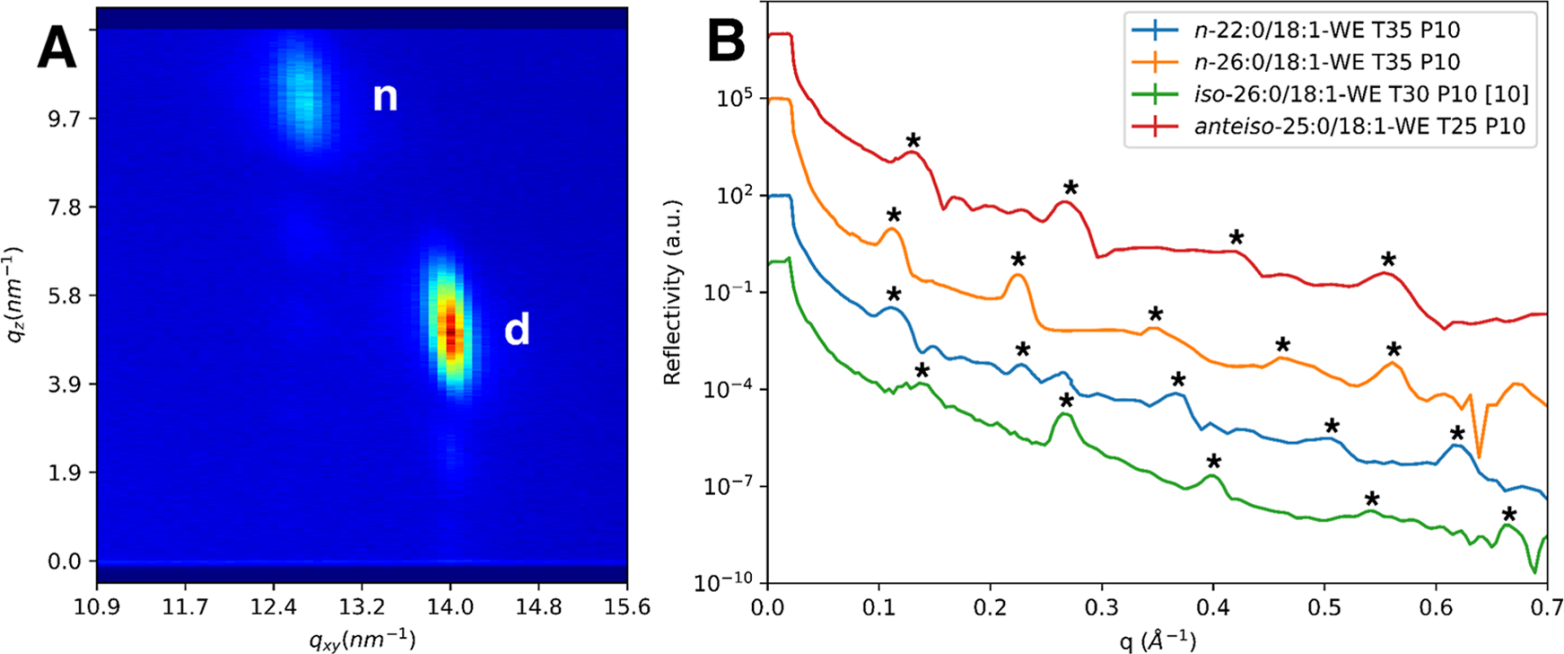
(A) *Anteiso*-25:0/18:1-WE GIXD pattern showing an *NNN* tilted phase. Note: d = degenerate peak (*11*, *1-1*); n = nondegenerate peak (*02*). (B) Comparison of XRR curves on series of wax ester samples (*anteiso*-25:0/18:1-WE in red). Repeating Bragg peaks are observed in them all (indicated by stars).

These GIXD results are complemented with XRR experiments. The previously studied *iso*-26:0/18:1-WE formed films composed of multilamellar structures at low surface pressures based on the presence of periodic Bragg peaks in the XRR curves.^10^ Here, we had the opportunity to investigate whether these structures were limited to the *iso*-26:0/18:1-WE, which films have distinct functional properties than the other WEs, or whether all WEs behave in a similar fashion regardless of branching pattern. We note that the new WEs studied herein, i.e. *anteiso*-25:0/18:1-WE, *n*-22:0/18:1-WE and *n*-26:0/18:1-WE, all formed similar multilamellar structures at the maximum surface pressures possible as those observed for the *iso*-branched species (see Figure 4B). Thus, branching does not seem to affect the baseline tendencies of these species to form multilamellar structures. Moreover, the lamellar distances for all of the species (45 ± 1 Å for *anteiso*-25:0/18:1-WE, 47 ± 1 Å for *iso*-26:0/18:1 WE, 50 ± 1 Å for *n*-22:0/18:1-WE and 56 ± 1 Å for *n*-26:0/18:1-WE) were in line with the values reported for meibum (50 Å)^11^ and the effects of the larger tilt angles observed in the GIXD-data translated into a shift toward a smaller lamellar distance which is reasonable. Altogether, this set of WEs allowed us to map the properties of the most abundant tear film WEs and highlight general features which would most probably be shared by the majority of WEs present in the TFLL as well.

We concluded in our recent work that while CEs make up a considerable portion of the TFLL, these are not ideal substrates for synchrotron or Langmuir trough studies since they are unable to form cohesive films. This does not mean that their contributions to the structure and function of the TFLL are unimportant. In fact, crystalline structures observed in the human TFLL in vivo may be important for its proper function and arise due to interactions driven by the intrinsic properties of CEs. On this note, the *iso*-26:0-CE studied earlier tended to form 3D crystallites when administered on an aqueous subphase.^10^ Interestingly, the *anteiso*-25:0-CE studied herein behaved in a similar fashion as shown by the Debye rings in Figure 5A. Thus, the properties of the studied CEs indicate that these could be important contributors to rigid and crystalline structures found in the TFLL. Simultaneously, it would in the future be important to address the interactions taking place between CEs and other species of the nonpolar lipid layer in order to investigate potential molecular assemblies that these may give rise to and assess their properties.

**Figure 5:**
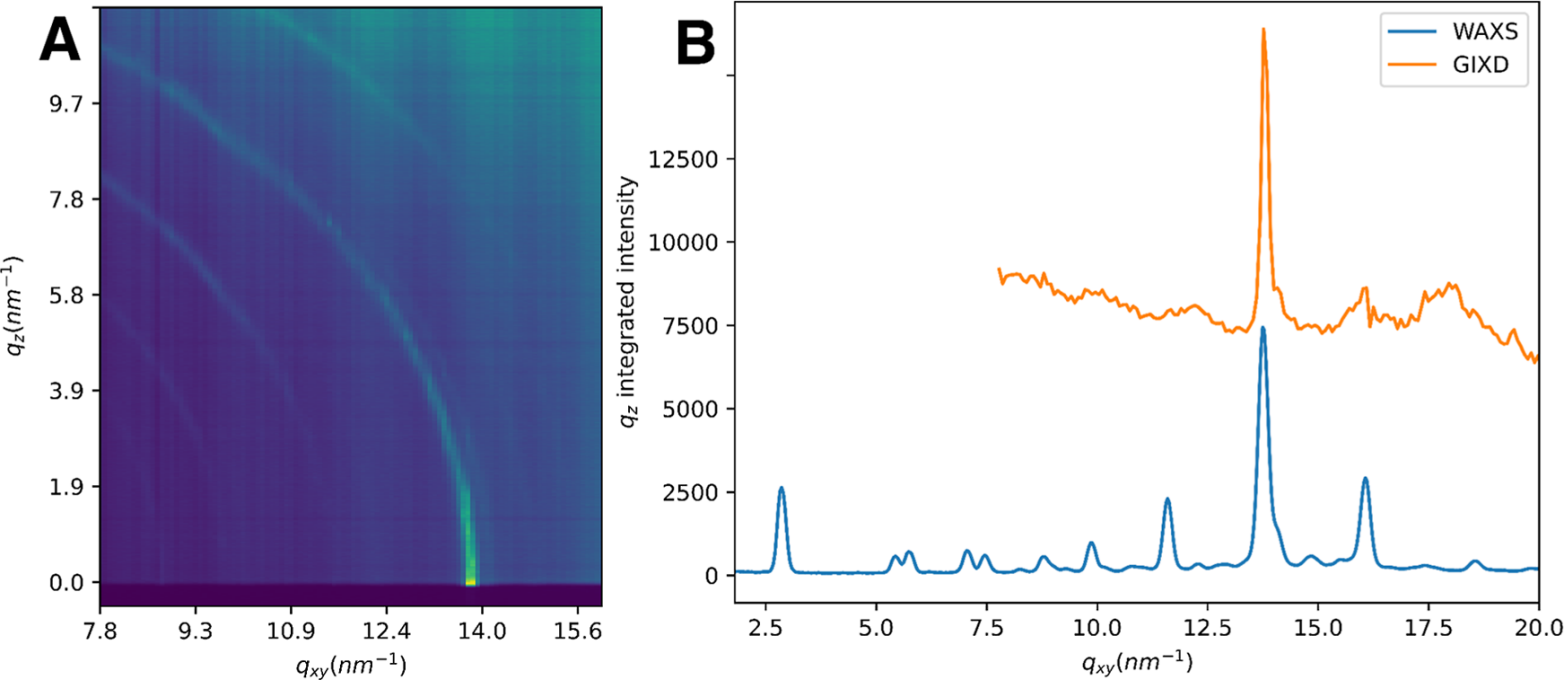
(A) *Anteiso*-25:0-CE GIXD image with Debye rings clearly visible, which indicates the presence of 3D crystalline structure. (B) Comparison of *anteiso*-25:0-CE’s integrated GIXD curve (in orange) with the WAXS curve^24^ (in blue) obtained in bulk state.

As can be assumed by the GIXD results, and in a similar fashion as for the previously studied *iso*-26:0-CE, the XRR data for the *anteiso*-25:0-CE did not show any clear structural features which could lead to quantitative insights. We concluded, similarly as for the *iso*-26:0-CE, that the rough surfaces generated by the 3D-crystallites would explain the strong and quick suppression of the XRR intensities as a function of *q*.

## Conclusions

Herein, we set out to continue our investigations on films formed by tear film lipids at the aqueous interface in order to identify their fundamental properties and potential contributions to the structure and function of the TFLL. We started by synthesizing and characterizing the most prominent OAHFA and type II DiE in the human tear film: the 32:1/18:1 OAHFA and 18:1/32:1/18:1-type II DiE. After profiling of core properties such as film behavior and melting points, we included these species alongside WEs and CEs with different branching patterns in GIXD and XRR studies to continue building on our understanding of the baseline features of distinct lipid classes. Several key observations on the molecular structure of tear film lipids could be made from the surface X-ray scattering data. For example, (1) all the lipid species studied (with the exception of *anteiso*-25:0-CE) have in-plane lattice parameters similar to those reported for meibum, (2) increasing the chain lengths of lipid species leads to shorter in-plane coherence lengths, (3) branching in WEs leads to tilt angles similar to those found in the most abundant OAHFA and type II DiEs, (4) *iso*/*anteiso*-branched CEs may contribute to rigid and crystalline domains observed in the TFLL, and, (5) the 32:1/18:1-OAHFA has a more complex film behavior than earlier witnessed for the OAHFA species with the possible multilamellar structures having lamellar distances similar to those found in WEs, which may be important for the adaptability of the lipid film.

Together, these results provide important molecular level insights into the fundamental properties and behavior of tear film lipids. In addition to identifying many similarities between the distinct tear film lipid classes, this study provides a solid baseline for studying and assessing the behavior of more complex compositions. The study of more complex compositions is where we intend to head in the future. Such studies will be important since the majority of the lipid species cannot be studied at ocular surface pressures due to film collapse which suggests that the interplay between these species is required in the natural environment. Therefore, we will in future move toward mapping the interactions taking place between different lipid classes and the arising effects on the stability, structure and function of the films which we consider to be critical for demystifying the structure and function of the TFLL.

## Supplementary Material



## Notes

The Supporting Information: insights on the origin of lipids studied, experimental details, synthesis and structural characterization discussion, synthetic protocols, characterization data, ^1^H and ^13^C NMR spectra of all synthesized compounds, supporting data from the synchrotron studies and surface pressure isotherms and BAM-images of the tear film lipids synthesized in this study (PDF)
