# Supplementary material for: One Step Closer to a Molecular Level Understanding of the Tear Film Lipid Layer by Surface X‑ray Scattering
Source: Langmuir. 2025 Aug 25;41(35):23353–61. doi: 10.1021/acs.langmuir.5c01920 (PMC12424177; doi:10.1021/acs.langmuir.5c01920)
Supplement: Supplementary file 1 [file la5c01920_si_001.pdf]

Supporting Information for:

"One Step Closer to a Molecular Level Understanding of the Tear Film Lipid Layer by Surface X-ray Scattering"

Ryan M. Trevorah,[1,ϕ] Henrik Stubb,[2,ϕ] Mira Viljanen,[1] Henrik Mäkinen,[1] Tuomo Viitaja,[2,3] Julia Sevón,[2] Oleg Konovalov,[4] Maciej Jankowski,[4] Arnaud Hemmerle,[5] Filip S. Ekholm,*[,2] and Kirsi J. Svedström*[,1]

[1] Department of Physics, University of Helsinki, P.O. Box 64, FI-00014 Helsinki, Finland

[2] Department of Chemistry, University of Helsinki, P.O. Box 55, FI-00014 Helsinki, Finland

[3] Ophthalmology, University of Helsinki and Helsinki University Hospital, Haartmaninkatu 8, FI-00290 Helsinki, Finland

[4] The European Synchrotron Radiation Facility – ESRF, 71 Avenue des Martyrs, CS 40220, Grenoble Cedex 9 38043, France

[5] Synchrotron SOLEIL, L'Orme des Merisiers, Départementale 128, 91190, Saint-Aubin, France

[ϕ] Equal contributions

Corresponding Author Contact: filip.ekholm@helsinki.fi, kirsi.svedstrom@helsinki.fi

Table of Contents:

1. **Supporting surface scattering results**
2. **Synthesis and structural characterization of tear film lipids**
3. **NMR spectra of synthesized compounds**
4. **Supporting Langmuir trough experiments**
5. **References**

1. Supporting surface scattering results

Table S1. variations in the structural parameters (lattice constants *a* and *b*, molecular tilt angle and the average in-plane coherence length *B*) as a function of temperature (T, given in °C) and pressure (P, given in mN/m). The lattice type (*NN* or *NNN*) is given in brackets.

Molecule and experiment details	*a* (Å)	*b* (Å)	Tilt angle (°)	*B*(in plane) (Å)
**32:1/18:1-OAHFA T30 P10 (*NN*)**	5.36 ± 0.03	8.54 ± 0.05	37 ± 0.5	45 ± 10
**32:1/18:1-OAHFA T30 P20 (*NN*)**	5.42 ± 0.03	8.42 ± 0.05	37 ± 0.5	85 ± 10
**32:1/18:1-OAHFA T30 P30 (*NN*)**	5.37 ± 0.03	8.41 ± 0.05	37 ± 0.5	80 ± 10
**32:1/18:1-OAHFA T30 P40 (*NN*)**	5.40 ± 0.03	8.38 ± 0.05	38 ± 0.5	90 ± 10
**32:1/18:1-OAHFA T35 P10 (*NN*)**	5.47 ± 0.03	8.67 ± 0.05	32 ± 0.5	60 ± 10
**32:1/18:1-OAHFA T35 P20 (*NN*)**	5.39 ± 0.03	8.73 ± 0.05	32 ± 0.5	160 ± 10
**32:1/18:1-OAHFA T35 P30 (*NN*)**	5.40 ± 0.03	8.69 ± 0.05	37 ± 0.5	130 ± 10
**32:1/18:1-OAHFA T35 P40 (*NN*)**	5.49 ± 0.03	8.50 ± 0.05	41 ± 0.5	330 ± 20
**18:1/32:1/18:1-type II DiE T35 P5 (*NN*)**	5.28 ± 0.03	9.17 ± 0.05	37 ± 0.5	250 ± 10
**18:1/32:1/18:1-type II DiE T35 P10(*NN*)**	5.26 ± 0.03	9.18 ± 0.05	37 ± 0.5	250 ± 10
***n*-26:0/18:1-WE T35 P10 (*NNN*)**	4.97 ± 0.03	8.24 ± 0.05	19 ± 0.5	70 ± 10
***n*-22:0/18:1-WE T35 P2 (*NNN*)**	5.01 ± 0.03	8.32 ± 0.05	17 ± 0.5	370 ± 10
***n*-22:0/18:1-WE T35 P5 (*NNN*)**	4.97 ± 0.03	8.42 ± 0.05	21 ± 0.5	75 ± 10
***n*-22:0/18:1-WE T35 P10 (*NNN*)**	5.00 ± 0.03	8.37 ± 0.05	20 ± 0.5	240 ± 10
***anteiso*-25:0/18:1-WE T25 P5 (*NNN*)**	5.04 ± 0.03	9.89 ± 0.05	39 ± 0.5	80 ± 10
***anteiso*-25:0/18:1-WE T25 P10 (*NNN*)**	5.02 ± 0.03	9.91 ± 0.05	40 ± 0.5	90 ± 10
**24:1-CE (did not crystallise)**	-	-	-	-

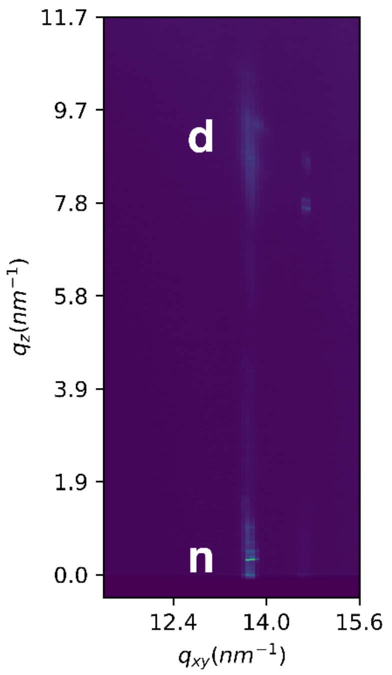

Figure S1: GIXD pattern of 18:1/32:1/18:1-type II DiE at 35°C, 5 mN/m. Two peaks can be observed, consistent with an *NN* lattice with the notes: *d* = degenerate peak (*11*, *1-1*); *n* = non-degenerate peak (*02*).

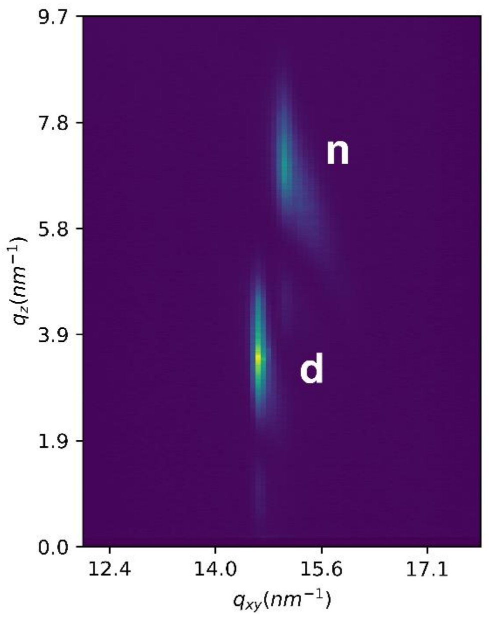

Figure S2: GIXD pattern of *n*-22:0/18:1-WE at 35°C, 10 mN/m. A next nearest neighbour tilted (*NNN*) lattice is identified with the peaks: *d* = degenerate peak (*11*, *1-1*); *n* = non-degenerate peak (*02*).

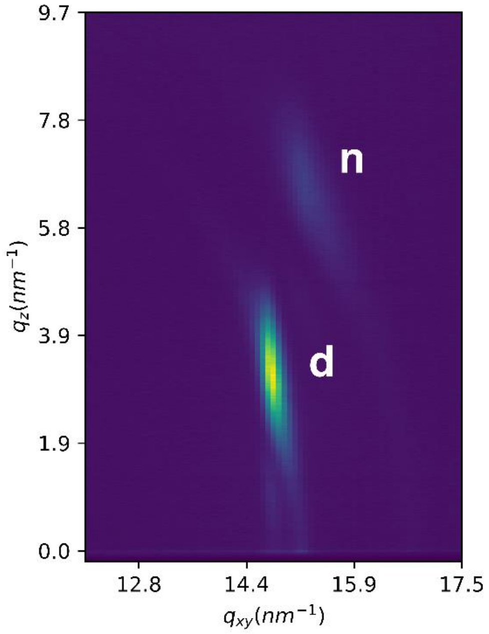

Figure S3: GIXD pattern of *n*-26:0/18:1-WE at 35°C, 10mN/m. An *NNN* lattice is identified with the peaks: *d* = degenerate peak (*11*, *1-1*); *n* = non-degenerate peak (*02*).

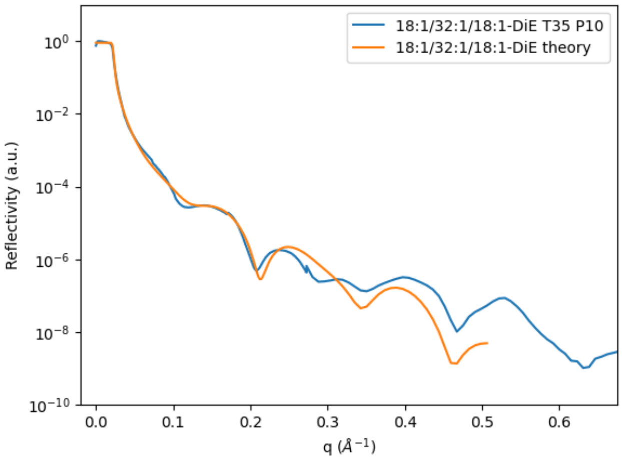

Figure S4. 18:1/32:1/18:1-type II DiE XRR data and best fit (fitted by EasyReflectometry) based by the model given in Table S3. This data was the most complex of those studied in this work and ultimately a 7-slab model was selected as the best fit.

Table S2. Slab model for 32:1/18:1-OAHFA (30 °C, 10 mN/m).

Refined Parameter	Refined value
Upper layer (tail)	
Thickness (Å)	54.9 ± 1.0
SLD (10^6 Å^{-2})	9.2 ± 0.5
Roughness (Å)	6.0 ± 0.5
Lower layer (head)	
Thickness (Å)	3.0 ± 1.0
SLD (10^6 Å^{-2})	13.3 ± 0.5
Roughness (Å)	2.5 ± 0.5
Total thickness (Å)	57.9 ± 2.0

Table S3. Slab model for 32:1/18:1-DiE (35 °C, 10 mN/m).

Refined Parameter	Refined value
7th layer	
Thickness (Å)	3.0 ± 1.0
SLD (10^6 Å^{-2})	9.4 ± 0.5
Roughness (Å)	3.9 ± 0.5
6th layer (tail)	
Thickness (Å)	17.6 ± 1.0
SLD (10^6 Å^{-2})	8.9 ± 0.5
Roughness (Å)	3.3 ± 0.5
5th layer	
Thickness (Å)	3.9 ± 1.0
SLD (10^6 Å^{-2})	9.4 ± 0.5
Roughness (Å)	3.0 ± 0.5
4th layer (tail)	
Thickness (Å)	24.0 ± 1.0
SLD (10^6 Å^{-2})	8.9 ± 0.5
Roughness (Å)	2.3 ± 0.5
3rd layer	
Thickness (Å)	8.0 ± 1.0
SLD (10^6 Å^{-2})	9.4 ± 0.5
Roughness (Å)	3.3 ± 0.5
2nd layer (tail)	
Thickness (Å)	23.0 ± 1.0
SLD (10^6 Å^{-2})	8.9 ± 0.5
Roughness (Å)	4.3 ± 0.5
1st layer	
Thickness (Å)	6.0 ± 1.0
SLD (10^6 Å^{-2})	9.4 ± 0.5
Roughness (Å)	5.35 ± 0.5

2. Synthesis and structural characterization of tear film lipids

Synthesis of 32:1/18:1-OAHFA and 18:1/32:1/18:1-Type II DiE (Supporting scheme 1): The starting materials selected for the synthesis of the 32:1-parent chain were docosanedioic acid and 9-decyn-1-ol. Docosanedioic acid was first converted into intermediate **2** (a suitable electrophilic species for a C-C bond forming reaction) by reducing the carboxylic acid functionalities with $LiAlH_4$, followed by monobromination employing aqueous HBr and cyclohexane in a biphasic system, and standard protection of the remaining hydroxy group as a THP ether. The yield of the reduction was moderate whereas the yields of the monobromination and THP-protection reactions were in the typical range. To obtain **1**, the hydroxy group in 9-decyn-1-ol was protected as a TIPS ether in excellent yield utilizing (*i*-Pr)$_3$SiCl as the silylation reagent and imidazole as the base. These two building blocks allow successful construction of the 32:1-parent chain base through a coupling reaction-reduction sequence. Moreover, the THP and TIPS ethers form an orthogonal protective group pair which was considered important for reaching the targeted tear film lipids.

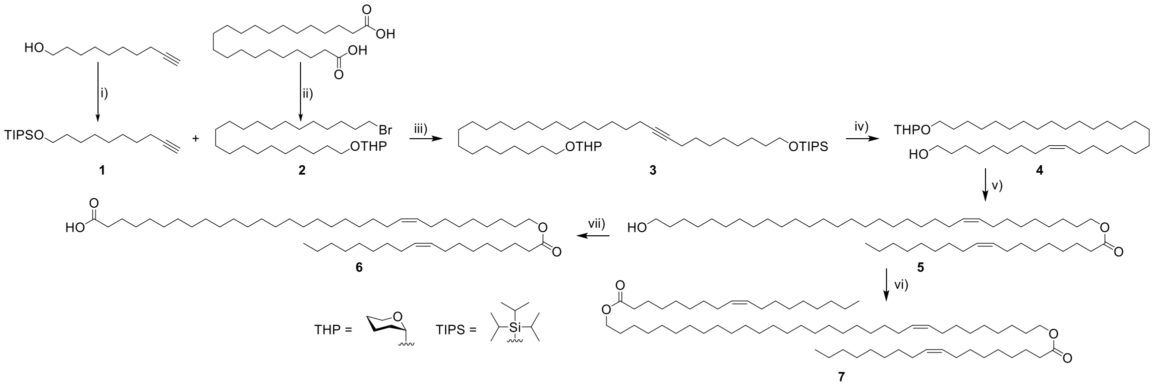

Supporting scheme 1. Overview of the total synthesis route leading to 32:1/18:1-OAHFA (**6**) and 18:1/32:1/18:1-Type II DiE (**7**). i) imidazole, chlorotriisopropylsilane, CH_2Cl_2, RT, 21 h, 95%; ii) 1) $LiAlH_4$, THF, RT $\rightarrow$ 66 °C, 15 h, 60%; 2) HBr, H_2O, cyclohexane, 80 °C, 10 h, 54%; 3) DHP, p-TsOH, CH_2Cl_2, RT, 17 h, 97%; iii) IAd-Cl, CuI, [(π-allyl)PdCl]$_2$, Cs_2CO_3, Et_2O:DMF 2:1, 40 °C, 20 h, 78%; iv) 1) TBAF, THF, RT, 4 h, 74%; 2) H_2, Lindlar catalyst, quinoline, benzene, RT, 1.5 h, >99%; v) 1) Oleic acid, EDC·HCl, DMAP, CH_2Cl_2, pyridine, RT, 23 h, 93%; 2) CSA, CH_3OH:THF 3:2, RT, 19 h, 94%; vi) Oleic acid, EDC·HCl, DMAP, CH_2Cl_2, pyridine, RT, 45 h, 97%; vii) Jones reagent, THF:acetone:EtOAc 1:2:1, RT, 1.5 h, 90%.

The C-C-bond forming reaction between the two key intermediates can be accomplished by various protocols. Here, we initially assessed protocols which feature the generation of an

acetylide anion from the terminal alkyne. In more detail, we used *n*-BuLi in THF at low temperatures for deprotonation and co-solvents such as HMPA or the more benign alternative DMPU, for acceleration of the S_N2-reaction which takes place at elevated temperatures after the addition of intermediate **2**. The yield of the reaction employing HMPA was good (69%) whereas the yield employing DMPU was suboptimal (48%). A yield of 69% is in the desired range, however, based on our previous success with the Sonogashira reaction[1] we decided to investigate whether it is a viable alternative to the more traditional C-C bond forming reactions. The Sonogashira reaction allows the C-C-bond formation to take place under milder reaction conditions thus potentially limiting the impact of the competing elimination reaction. Indeed, our recently employed Sonogashira protocol was found to be more efficient also in the current case thereby leading to an 78% isolated yield of the orthogonally protected alkyne **3**.

Following the successful C-C bond formation the silyl ether was removed to unmask the temporarily protected hydroxy group. For this purpose, we used the reagent TBAF as fluoride anions have a strong affinity for silicon. The deprotection proceeded smoothly in a 74% yield. At this stage, we decided to perform the selective reduction of the alkyne to yield a *Z*-alkene. To accomplish this, the reaction was performed in an autoclave with Lindlar catalyst, benzene as the solvent, and a H_2-pressure of 1 atmosphere. This gave **4** in a quantitative yield with excellent *Z*-selectivity while avoiding over-reduction. To estimate the *E*/*Z*-isomer ratios in the product, we utilized the power of a quantum mechanical spectral analysis (QMSA) tool (see Figure S5), which has been validated in several studies, e.g. by Tiainen et al.[2]. In more detail, the ratios of the *E*/*Z*-isomers isomer could be quantified based on the ^{1}H-NMR spectral data utilizing the intensities observed in the alkene region. The coupling constants of the *Z*-isomer have previously been determined to be $J_{9,11}$ = –1.6, $J_{9,8}$ = 7.3, and $J_{9,10}$ = 10.8 Hz, (the coupling constants for H-9 and H-10 are the same) and the *E*-isomer is expected to have similar values, except for the coupling constant between H-9 and H-10 which should be considerably higher, ~ 14–17 Hz range. Our initial assessment revealed that coupling constant values of $J_{9,11}$ = –1.8, $J_{9,8}$ = 6.7, and $J_{9,10}$ = 15.0 Hz resulted in a good match between the observed and calculated spectral intensities and could be utilized to consistently assess the ratios of *E*/*Z*-isomers in the product mixture. Under optimal reaction conditions, *Z*/*E*-ratios of > 98:2 (> 98% of the wanted *Z*-isomer) could be achieved which we consider to be an excellent finding. This is because we

noticed that this reaction is sensitive to the reaction conditions employed. For example, a 30-minute increase in the reaction time gave an *E*/*Z*-ratio of ~ 10:90 whereas the use of the more benign solvent toluene resulted in an *E*/*Z*-ratio of ~ 14:86.

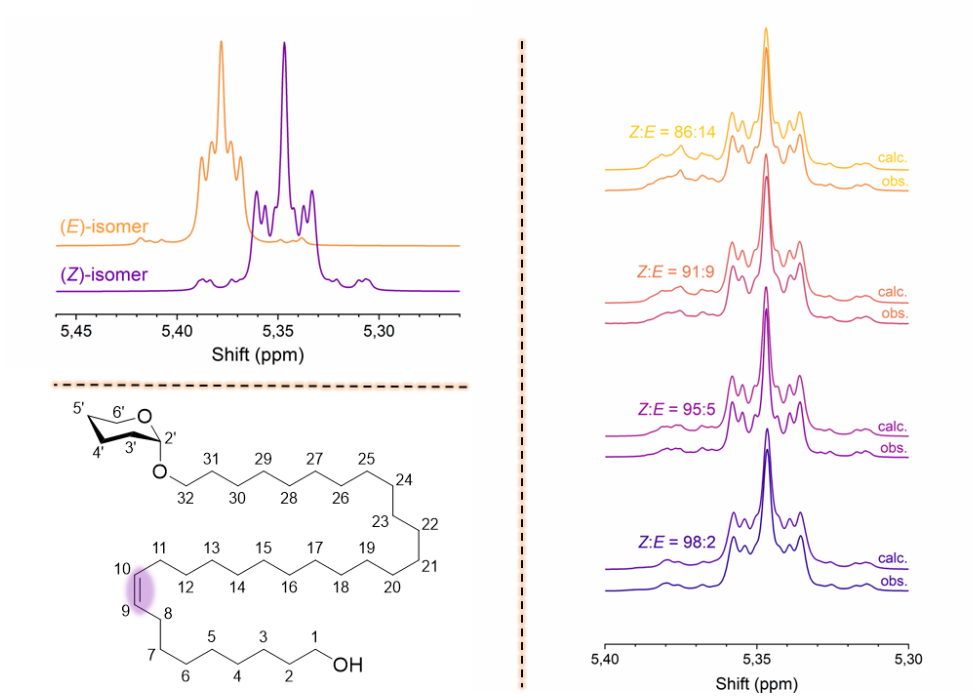

Figure S5. Excerpt of the QMSA tools applied in characterization of NMR spectra with special highlights from the characterization of the alkene region in key intermediate **4**. **Top left panel:** Simulation of the pure (*Z*)- (bottom, purple) and (*E*)-isomers (top, orange) reveal notable differences in chemical shifts and coupling constants. **Bottom left panel:** The numbering used for **4** is shown with the double bond marked in purple. **Right panel:** Calculated spectra (top) are shown in pairs together with the corresponding observed spectra (bottom) from different reactions tested indicating the accuracy of our approach to determining the *E*/*Z*-ratios of the product.

With access to key intermediate **4**, we proceeded with an esterification of the unmasked hydroxy group. Utilizing EDC·HCl as the coupling reagent and oleic acid as the carboxylic acid, a yield of 93% could be obtained in the esterification. For construction of the 32:1/18:1-OAHFA (**6**), the THP ether was deprotected under acidic conditions employing CSA and the resulting alcohol **5** oxidized with Jones reagent in an 85% overall yield over two steps. For synthesis of the 18:1/32:1/18:1-type II DiE (**7**), the alcohol **5** was subjected to an esterification reaction utilizing EDC·HCl and oleic acid in an excellent yield.

In addition to the TFLL-lipids synthesized for this study specifically (discussed above and reported on in the experimental section below), several TFLL lipids synthesized in our earlier work were studied by synchrotron techniques here. Table S4 summarizes the origin of the lipids studied herein and in our earlier work[3] and provides references from which synthetic protocols and characterization data for these compounds can be found. This table complements the information given in Figure 1 in the main manuscript which describes which TFLL-lipids are reported on here for the first time. For the previously synthesized lipid compounds, their identity was assessed prior to our studies here and matched the characterization data reported by us previously.

Table S4. A table of the lipids studied herein. The table includes the abbreviated name, molecular structure and a reference to the original publication in which synthetic protocols and characterization data can be found. For the commercially available lipid, the table includes the source from which it was acquired.

Abbreviation	Molecular structure	Reference/supplier
**29:1/18:1-OAHFA**	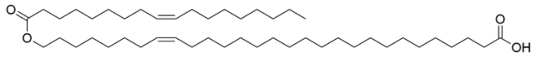	*Colloids Surf. B Biointerfaces* **2023**, 223, 113145.[4]
**32:1/18:1-OAHFA**	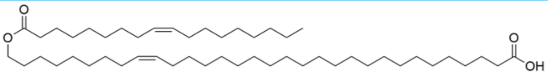	Reported herein
**18:1/29:1/18:1-type II DiE**	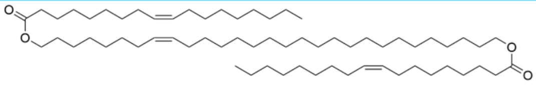	*Colloids Surf. B Biointerfaces* **2023**, 223, 113145.[4]
**18:1/32:1/18:1-type II DiE**	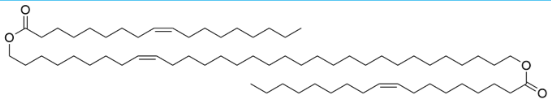	Reported herein
***n*-26:0/18:1-WE**	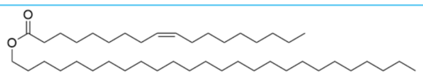	*Colloids Surf. B Biointerfaces* **2022**, 214, 112429.[5]
***n*-22:0/18:1-WE**	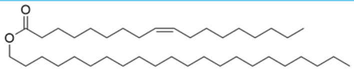	Commercial, Larodan AB
***iso*-26:0/18:1-WE**	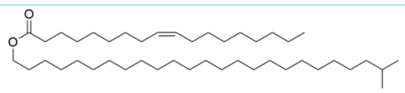	*Colloids Surf. B Biointerfaces* **2022**, 214, 112429.[5]
***anteiso*-25:0/18:1-WE**	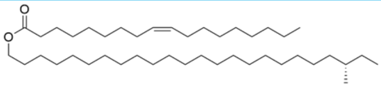	*J. Nat. Prod.* **2024**, 87, 954.[1]
***iso*-26:0-CE**	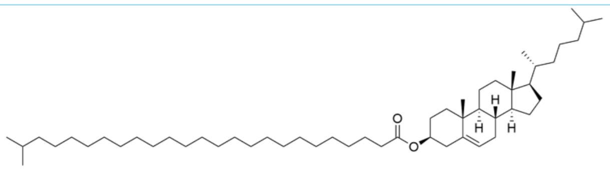	*Colloids Surf. B Biointerfaces* **2022**, 214, 112429.[5]
***anteiso*-25:0-CE**	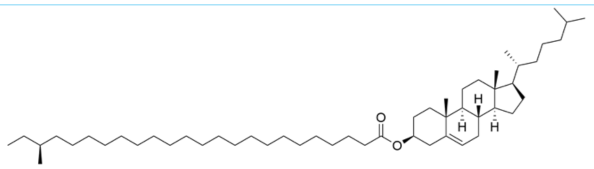	*J. Nat. Prod.* **2024**, 87, 954.[1]

Experimental section: The starting materials and reagents were purchased from commercial sources (Acros organics, Honeywell, Fluorochem, Merck, Avantor) and used as obtained without further purification. Dry solvents were acquired by the use of a VAC vacuum solvent purification system when required. All reactions containing moisture- or air-sensitive reagents were carried out under an argon atmosphere. TLC was performed on aluminum sheets precoated with silica gel 60 F254 (Merck) and spots were visualized by spraying the plates with a 5:1 MeOH/H_2SO_4-solution followed by charring. Flash chromatography was carried out on silica gel 40 (40–63 µm, Merck). NMR spectra were recorded on a Bruker Avance III instrument operating at 500 MHz (^{1}H = 499.82 MHz, ^{13}C = 125.68 MHz). The probe temperature was kept at 25 °C. The NMR spectra were processed using the Bruker TopSpin 4.3.0 software, and the quantum mechanical spectral analysis (QMSA) was performed with the Chemadder software (Spin Discoveries Inc., Kuopio, Finland). Isomer ratios were confirmed by quantitative QMSA (qQMSA), following methods developed by Tiainen et al. that have a mean absolute error of <10%. [2] The chemical shifts are expressed on the ppm scale using tetramethylsilane (TMS; δ_H 0 ppm; δ_C 0 ppm) as the internal reference. Coupling constants are given in Hz and provided only once when first encountered. The coupling patterns are given as singlet (s), doublet (d), triplet (t), multiplet (m), etc. A standard set of 1D (^{1}H and ^{13}C) and 2D (DQF-COSY, Ed-HSQC, HMBC) NMR experiments were recorded with pulse sequences provided by the instrument manufacturer. High resolution mass spectra (HRMS) were recorded on a Bruker micro Q-TOF mass spectrometer with ESI (electrospray ionization) operated in positive mode, or ThermoScientific Q Exactive HF Orbitrap MS with heated-electrospray ionization (H-ESI) operated in positive or negative (32:1/18:1-OAHFA) mode. Compounds with a low *m/z*-ratio were analyzed by GC−MS on a Bruker Scion 456-GC (column: DB-5ms, 5% phenylmethylsiloxane, 30 m × 0.25 mm i.d.; carrier gas: He, 1.3 mL/min; pressure: 98 kPa; Temperature program: 135 °C (4 min), then 5 °C/min to 280 °C (10 min)) with single quadrupole mass analyzer and electron ionization (EI, 70 eV). Melting points were determined on a Mettler Toledo MP50 (Columbus, OH, USA) melting point device with an accuracy of 0.1 °C. Samples for melting point determination were prepared into 1.5–1.6 mm capillary tubes (Fortuna WGG, Germany) and heated at a rate of 0.5 °C/min. Melting point measurements were done in triplicate.

1-(Tri-isopropylsilyloxy)dec-9-yne (1). To an ice bath-cooled solution of 9-decyn-1-ol (2.523 g, 16.36 mmol, 1.0 equivalents) in dry CH_2Cl_2 (35 mL) were added imidazole (2.230 g, 32.76 mmol, 2.0 equivalents) and chlorotriisopropylsilane (3.83 mL, 17.98 mmol, 1.1 equivalents). The ice bath was removed, and the solution was stirred at RT under argon for 21 h. The reaction mixture was then diluted with a saturated aqueous solution of $NaHCO_3$ (50 mL), and the aqueous phase was extracted with CH_2Cl_2 (2 × 40 mL). The combined organic phases were dried over anhydrous Na_2SO_4, filtered, and evaporated under reduced pressure. The crude product was purified by silica gel flash chromatography (*n*-hexane:EtOAc 95:5) and dried *in vacuo* to obtain a colorless oil (4.804 g, 95%). R_f = 0.23 (*n*-hexane:EtOAc 98:2).

^{1}H NMR (499.82 MHz, $CDCl_3$, 25 °C): δ_H 3.67 (2H, t, $J_{1,2}$ = 6.6 Hz, H-1), 2.18 (2H, dt, $J_{8,10}$ = 2.6, $J_{8,7}$ = 7.2 Hz, H-8), 1.93 (1H, t, H-10), 1.53 (2H, tt, $J_{2,3}$ = 8.1 Hz, H-2), 1.52 (2H, tt, $J_{7,6}$ = 8.2 Hz, H-7), 1.39 (2H, tt, $J_{6,5}$ = 6.9 Hz, H-6), 1.34 (2H, m, H-3), 1.33−1.28 (4H, m, H-4, H-5), 1.09 (3H, qq, $J_{CH,CH3}$ = 7.6 Hz, $OSi(C\underline{H}(CH_3)_2)_3$), and 1.06 (18H, d, $OSi(CH(C\underline{H}_3)_2)_3$) ppm.

^{13}C NMR (125.68 MHz, $CDCl_3$, 25 °C): δ_C 84.8 (C9), 68.0 (C10), 63.5 (C1), 33.0 (C2), 29.3 (C4), 29.1 (C5), 28.7 (C6), 28.5 (C7), 25.8 (C3), 18.4 (C8), 18.0 ($OSi(CH(\underline{C}H_3)_2)_3$), and 12.0 ($OSi(\underline{C}H(CH_3)_2)_3$) ppm.

GC-MS t_R = 17.56 min: 310.2 (0.2) [M^+, $C_{19}H_{38}OSi$], 268.3 (20), 267.2 (94), 225.5 (77), 197.1 (45), 183.1 (21), 171.1 (38), 157.1 (68), 145.0 (52), 143.0 (100).

1,22-Docosanediol. To an ice bath-cooled solution of docosanedioic acid (1.000 g, 2.70 mmol, 1.0 equivalents) in dry THF (70 mL) was added $LiAlH_4$ (512 mg, 13.49 mmol, 5.0 equivalents) in small portions. The ice bath was removed, the solution was warmed to RT, then refluxed (66 °C) for 15 h. The resulting suspension was cooled to RT, then cooled in an ice bath, and wet Na_2SO_4 was added in small portions until evolution of H_2 ceased. Concentrated HCl (37%, 5 mL) and THF (30 mL) were added, and the suspension was stirred at RT for 3 h. The resulting clear solution was filtered through diatomaceous earth. The remaining solids and the diatomaceous earth were washed with THF (100 mL), and the filtrate was evaporated under reduced pressure. The crude product was redissolved in toluene (50 mL), and a hot extraction was performed with 1 M KOH (15 mL), then with 1 M HCl (15 mL). The extractions were repeated twice, and the title compound was crystallized from the cold toluene phase and dried *in vacuo* to obtain a white powder (553 mg, 60%).

^{1}H NMR (499.82 MHz, MeOD:$CDCl_3$ 1:1, 25 °C): δ_H 3.56 (4H, t, $J_{1,2;22,21}$ = 6.8 Hz, H-1, H-22), 1.55 (4H, tt, $J_{2,3;21,20}$ = 7.3 Hz, H-2, H-21), 1.33 (4H, m, H-3, H-20), and 1.32−1.26 (34H, 1-OH, 22-OH, H-4−H19) ppm.

^{13}C NMR (125.68 MHz, MeOD:$CDCl_3$ 1:1, 25 °C): δ_C 63.1 (C1, C22), 32.8 (C2, C21), 29.7−29.4 (C4−C19), and 25.8 (C3, C20) ppm.

HRMS m/z 343.3573 $[M + H]^+$ (calculated for $C_{22}H_{47}O_2$, 343.2571).

22-Bromo-1-docosanol. 1,22-Docosanediol (1.006 g, 2.94 mmol, 1.0 equivalents), cyclohexane (37 mL) and HBr (48% in H_2O, 10.0 mL, 88.39 mmol, 30.1 equivalents) were refluxed (81 °C) and stirred vigorously for 10 h. The reaction mixture was cooled to RT and a saturated aqueous solution of $NaHCO_3$ (100 mL) was added to reach a pH of 8. The aqueous phase was extracted with CH_2Cl_2 (5 × 30 mL). The combined organic phases were washed with brine (30 mL), dried over anhydrous Na_2SO_4, filtered, and evaporated under reduced pressure. The crude product was purified by silica gel flash chromatography (*n*-hexane:EtOAc 4:1) and dried *in vacuo* to obtain a white solid (644 mg, 54%). R_f = 0.63 (*n*-hexane:EtOAc 2:1).

^{1}H NMR (499.82 MHz, $CDCl_3$, 25 °C): δ_H 3.64 (2H, dt, $J_{1,1\text{-OH}}$ = 4.8, $J_{1,2}$ = 6.7 Hz, H-1), 3.40 (2H, t, $J_{22,21}$ = 6.9 Hz, H-22), 1.85 (2H, tt, $J_{21,20}$ = 7.5 Hz, H-21), 1.67 (2H, tt, $J_{2,3}$ = 7.4 Hz, H-2), 1.42 (2H, tt, $J_{20,19}$ = 7.3 Hz, H-20), 1.35 (2H, m, H-3), 1.31 (2H, m, H-19), 1.30−1.23 (30H, m, H-4−H-18), and 1.22 (1H, t, 1-OH) ppm.

^{13}C NMR (125.68 MHz, $CDCl_3$, 25 °C): δ_C 63.1 (C1), 34.1 (C22), 32.9 (C21), 32.8 (C2), 29.7−29.4 (C4−C18), 28.8 (C19), 28.2 (C20), and 25.8 (C3) ppm.

22-Bromo-1-(2'-tetrahydropyranyloxy)docosane (2). A solution of 22-bromo-1-docosanol (639 mg, 1.58 mmol, 1.0 equivalents), pyridinium *p*-toluenesulfonate (40 mg, 0.16 mmol, 0.1 equivalents), and 3,4-dihydro-2*H*-pyran (265 mg, 3.15 mmol, 2.0 equivalents) in dry CH_2Cl_2 (50 mL) were stirred at RT under argon for 17 h. H_2O (40 mL) was added and the aqueous phase was extracted with CH_2Cl_2 (2 × 40 mL). The combined organic phases were dried over anhydrous Na_2SO_4, filtered, and evaporated under reduced pressure. The crude product was purified by silica gel flash chromatography (*n*-hexane:EtOAc 95:5) and dried *in vacuo* to obtain a white solid (737 mg, 97%). R_f = 0.51 (*n*-hexane:EtOAc 9:1).

^{1}H NMR (499.82 MHz, $CDCl_3$, 25 °C): δ_H 4.58 (1H, dd, $J_{2',3'a}$ = 2.8, $J_{2',3'b}$ = 4.6 Hz, H-2'), 3.87 (1H, ddd, $J_{6'a,5'b}$ = 3.0, $J_{6'a,5'a}$ = 7.3, $J_{6'a,6'b}$ = –11.3 Hz, H-6'a), 3.73 (1H, dt, $J_{1a,2}$ = 6.9, $J_{1a,1b}$ = −9.5 Hz,

H-1a), 3.50 (1H, m, H-6'b), 3.41 (2H, t, $J_{22,21}$ = 6.9 Hz, H-22), 3.38 (1H, dt, $J_{1b,2}$ = 6.8 Hz, H-1b), 1.85 (2H, tt, $J_{21,20}$ = 7.6 Hz, H-21), 1.83 (1H, ddddd, $J_{4'a,3'a}$ = 9.2, $J_{4'a,4'b}$ = −12.0 Hz, H-4'a), 1.71 (1H, dddd, $J_{3'a,4'b}$ = 4.1, $J_{3'a,3'b}$ = −13.1 Hz, H-3'a), 1.59 (2H, ddt, $J_{2,3}$ = 7.2 Hz, H-2), 1.58−1.48 (4H, m, H-3'b, H-4'b, H-5'a, H-5'b), 1.42 (2H, tt, $J_{20,19}$ = 7.4 Hz, H-20), 1.35 (2H, m, H-3), and 1.33−1.23 (32H, m, H-3−H-18) ppm.

^{13}C NMR (125.68 MHz, $CDCl_3$, 25 °C): δ_C 98.9 (C2'), 67.7 (C1), 62.4 (C6'), 34.1 (C22), 32.9 (C21), 30.8 (C3'), 29.8 (C2), 29.7−29.5 (C-4−C-18), 28.8 (C19), 28.2 (C20), 26.3 (C3), 25.5 (C5'), and 19.7 (C4') ppm.

HRMS m/z 511.3122 $[M + Na]^+$ (calculated for $C_{27}H_{53}BrO_2Na$, 511.3121).

1-Tri-isopropylsilyloxy-32-(2'-tetrahydropyranyloxy)dotriacont-9-yne (3). 1,3-Bis-(1-adamantyl)imidazolium chloride (IAd-Cl, 93 mg, 0.25 mmol, 15 mol%), CuI (71 mg, 0.37 mmol, 22 mol%), $[(\pi\text{-allyl})PdCl]_2$ (47 mg, 0.13 mmol, 8 mol%), and Cs_2CO_3 (766 mg, 2.35 mmol, 1.4 equivalents) were placed in a Schlenk tube, which was thrice evacuated (<1 mbar) and backfilled with argon. Using standard Schlenk techniques, dry and degassed (freeze-pump-thaw method) DMF (3 mL) was added. To the yellow suspension were added alkyne **1** (664 mg, 2.14 mmol, 1.3 equivalents), and alkyl halide **2** (816 mg, 1.67 mmol, 1.0 equivalents) in a solution of dry and degassed Et_2O (6 mL), resulting in a black mixture. Any resulting needle puncture holes were covered with parafilm. The suspension was heated to 40 °C and stirred for 20 h, and then quenched by addition of a saturated aqueous solution of NH_4Cl (20 mL) and Et_2O (50 mL). The aqueous phase was extracted with Et_2O (3 × 60 mL). The combined organic phases were dried over anhydrous Na_2SO_4 and filtered through diatomaceous earth. The diatomaceous earth was washed with Et_2O (50 mL), and the filtrate was evaporated under reduced pressure. The crude product was purified by silica gel flash chromatography (*n*-hexane:EtOAc 98:2 $\rightarrow$ 95:5) and dried *in vacuo* to obtain an orange oil (932 mg, 78%, purity: ~85%). R_f = 0.53 (*n*-hexane:EtOAc 9:1).

^{1}H NMR (499.82 MHz, $CDCl_3$, 25 °C): δ_H 4.58 (1H, dd, $J_{2',3'a}$ = 2.8, $J_{2',3'b}$ = 4.4 Hz, H-2'), 3.87 (1H, ddd, $J_{6'a,5'a}$ = 2.7, $J_{6'a,5'b}$ = 7.9, $J_{6'a,6'b}$ = −11.4 Hz, H-6'a), 3.73 (1H, dt, $J_{32a,31}$ = 6.9, $J_{32a,32b}$ = −9.6 Hz, H-32a), 3.66 (2H, t, $J_{1,2}$ = 6.7 Hz, H-1), 3.50 (1H, ddd, $J_{6'b,5'a}$ = 4.1, $J_{6'b,5'b}$ = 5.8 Hz, H-6'b), 3.38 (1H, dt, $J_{32b,31}$ = 6.7 Hz, H-32b), 2.13 (4H, each tt, $J_{8,11}$ = −2.3, $J_{8,7}$ = 7.1, $J_{11,12}$ = 7.1 Hz, H-8, H-11), 1.83 (1H, ddddd, $J_{4'a,5'b}$ = 3.0, $J_{4'a,3'b}$ = 4.0, $J_{4'a,5'a}$ = 8.6, $J_{4'a,3'a}$ = 9.2, $J_{4'a,4'b}$ = −12.4 Hz, H-

4’a), 1.71 (1H, dddd, $J_{3'a,4'b}$ = 3.6, $J_{3'a,3'b}$ = −13.1 Hz, H-3’a), 1.59 (2H, tt, $J_{31,30}$ = 7.1 Hz, H-31), 1.58−1.50 (6H, m, H-2, H-3’b, H-4’b, H-5’a, H-5’b), 1.47 (4H, each tt, $J_{12,13}$ = 6.7, $J_{7,6}$ = 6.8 Hz, H-7, H-12), 1.39−1.29 (8H, m, H-3, H-6, H-13, H-30), 1.28−1.23 (36H, m, H-4, H-5, H-14−H-29), 1.09 (3H, qq, $J_{CH,CH3}$ = 7.6 Hz, OSi(C<u>H</u>$(CH_3)_2)_3$), and 1.06 (18H, d, OSi(CH(C<u>H</u>$_3)_2)_3$) ppm.

^{13}C NMR (125.68 MHz, $CDCl_3$, 25 °C): δ_C 98.9 (C2’), 80.3−80.2 (C9, C10), 67.7 (C32), 63.5 (C1), 62.3 (C6’), 33.1 (C2), 30.8 (C3’), 29.8 (C31), 29.7−29.4 (C4, C5, C14−C29), 29.2 (C7, C12), 28.9−28.8 (C6, C13), 26.3 (C30), 25.8 (C3), 25.5 (C5’), 19.7 (C4’), 18.8 (C8, C11), 18.0 (OSi(CH(<u>C</u>H$_3)_2)_3$), and 12.0 (OSi(<u>C</u>H$(CH_3)_2)_3$) ppm.

HRMS m/z 741.6512 $[M + Na]^+$ (calculated for $C_{46}H_{90}O_3SiNa$, 741.6551).

32-(2’-Tetrahydropyranyloxy)dotriacont-9-yn-1-ol. To alkyne **3** (768 mg, 1.07 mmol, 1.0 equivalents) dissolved in dry THF (15 mL) was added TBAF (1.0 mol/L in THF, 3.2 mL, 3.20 mmol, 3.0 equivalents) at 0 °C. The solution was stirred at RT under argon for 4 h, then H_2O (20 mL) was added. The aqueous phase was extracted with EtOAc (3 × 40 mL), and the combined organic phases were washed with brine (20 mL), dried over anhydrous Na_2SO_4, filtered, and evaporated under reduced pressure. The crude product was purified by silica gel flash chromatography (*n*-hexane:EtOAc 4:1) and dried *in vacuo* to obtain a white solid (446 mg, 74%). R_f = 0.32 (*n*-hexane:EtOAc 4:1).

^{1}H NMR (499.82 MHz, $CDCl_3$, 25 °C): δ_H 4.58 (1H, dd, $J_{2',3'a}$ = 2.8, $J_{2',3'b}$ = 4.5 Hz, H-2’), 3.87 (1H, ddd, $J_{6'a,5'a}$ = 2.7, $J_{6'a,5'b}$ = 7.9, $J_{6'a,6'b}$ = −11.4 Hz, H-6’a), 3.73 (1H, dt, $J_{32a,31}$ = 6.9, $J_{32a,32b}$ = −9.6 Hz, H-32a), 3.64 (2H, dt, $J_{1,1\text{-}OH}$ = 5.2, $J_{1,2}$ = 6.6 Hz, H-1), 3.50 (1H, ddd, $J_{6'b,5'a}$ = 4.1, $J_{6'b,5'b}$ = 5.8 Hz, H-6’b), 3.38 (1H, dt, $J_{32b,31}$ = 6.7 Hz, H-32b), 2.13 (4H, each tt, $J_{8,11}$ = −2.7, $J_{8,7;11,12}$ = 7.4 Hz, H-8, H-11), 1.83 (1H, ddddd, $J_{4'a,5'b}$ = 3.0, $J_{4'a,3'b}$ = 4.0, $J_{4'a,5'a}$ = 8.6, $J_{4'a,3'a}$ = 9.2, $J_{4'a,4'b}$ = −12.4 Hz, H-4’a), 1.71 (1H, dddd, $J_{3'a,4'b}$ = 3.6, $J_{3'a,3'b}$ = −13.1 Hz, H-3’a), 1.59 (2H, tt, $J_{31,30}$ = 7.1 Hz, H-31), 1.58−1.49 (6H, m, H-2, H-3’b, H-4’b, H-5’a, H-5’b), 1.47 (4H, each tt, $J_{7,6;12,13}$ = 7.4 Hz, H-7, H-12), 1.40−1.29 (8H, m, H-3, H-6, H-13, H-30), 1.28−1.24 (36H, m, H-4, H-5, H-14−H-29), 1.23 (1H, t, 1-OH) ppm.

^{13}C NMR (125.68 MHz, $CDCl_3$, 25 °C): δ_C 98.9 (C2’), 80.3−80.2 (C9, C10), 67.7 (C32), 63.1 (C1), 62.3 (C6’), 32.8 (C2), 30.8 (C3’), 29.8 (C31), 29.7−29.1 (C4, C5, C7, C12, C14−C29), 28.9−28.8 (C6, C13), 26.3 (C30), 25.7 (C3), 25.5 (C5’), 19.7 (C4’), and 18.8 (C8, C11) ppm.

HRMS m/z 585.5247 $[M + Na]^+$ (calculated for $C_{37}H_{70}O_3Na$, 585.5217).

(9*Z*)-32-(2'-Tetrahydropyranyloxy)dotriacont-9-en-1-ol (4). In a Parr reactor, 32-(2'-tetrahydropyranyloxy)dotriacont-9-yn-1-ol (232 mg, 0.41 mmol, 1.0 equivalents), Pd 5%/$CaCO_3$/Pb (Lindlar catalyst, 115 mg, 0.5 mass-equivalents), and quinoline (436 µL, 3.69 mmol, 9.0 equivalents) were dissolved in dry benzene (56 mL). The suspension was purged with N_2 and then stirred vigorously under H_2 (1 bar) at RT for 1.5 h. The suspension was filtered through diatomaceous earth. The diatomaceous earth was washed with EtOAc (200 mL), and the filtrate was evaporated under reduced pressure. The crude product was purified by silica gel flash chromatography (*n*-hexane:EtOAc 4:1) and dried *in vacuo* to obtain a white solid (235 mg, >99%, *Z*/*E* ratio > 98:2). R_f = 0.57 (*n*-hexane:EtOAc 2:1).

^{1}H NMR (499.82 MHz, $CDCl_3$, 25 °C): δ_H 5.35 (1H, dtt, $J_{9,11}$ = −1.6, $J_{9,8}$ = 7.3, $J_{9,10}$ = 10.8 Hz, H-9), 5.34 (1H, dtt, $J_{10,8}$ = −1.6, $J_{10,11}$ = 7.3 Hz, H-10), 4.58 (1H, dd, $J_{2',3'a}$ = 2.8, $J_{2',3'b}$ = 4.6 Hz, H-2'), 3.97 (1H, ddd, $J_{6'a,5'a}$ = 2.7, $J_{6'a,5'b}$ = 7.9, $J_{6'a,6'b}$ = −11.4 Hz, H-6'a), 3.73 (1H, dt, $J_{32a,31}$ = 6.9, $J_{32a,32b}$ = −9.6 Hz, H-32a), 3.64 (2H, dt, $J_{1,1\text{-}OH}$ = 4.4, $J_{1,2}$ = 6.5 Hz, H-1), 3.50 (1H, ddd, $J_{6'b,5'a}$ = 4.1, $J_{6'b,5'b}$ = 5.8 Hz, H-6'b), 3.38 (1H, dt, $J_{32b,31}$ = 6.7 Hz, H-32b), 2.01 (4H, each ddt, $J_{8,7;11,12}$ = 6.6 Hz, H-8, H-11), 1.83 (1H, ddddd, $J_{4'a,5'b}$ = 3.1, $J_{4'a,3'b}$ = 3.8, $J_{4'a,5'a}$ = 8.6, $J_{4'a,3'a}$ = 9.2, $J_{4'a,4'b}$ = −12.1 Hz, H-4'a), 1.71 (1H, dddd, $J_{3'a,4'b}$ = 3.7, $J_{3'a,3'b}$ = −12.8 Hz, H-3'a), 1.59 (2H, ddt, $J_{31,30}$ = 7.1 Hz, H-31), 1.58−1.49 (6H, m, H-2, H-3'b, H-4'b, H-5'a, H-5'b), 1.38−1.29 (8H, m, H-3, H-7, H-12, H-30), and 1.28−1.23 (41H, m, 1-OH, H-4−H-6, H-14−H-29) ppm.

^{13}C NMR (125.68 MHz, $CDCl_3$, 25 °C): δ_C 130.0 (C9), 129.8 (C10), 98.9 (C2'), 67.7 (C32), 63.1 (C1), 62.3 (C6'), 32.8 (C2), 30.8 (C3'), 29.8 (C31), 29.8−29.2 (C4−C7, C12−C29), 27.2 (C8, C11), 26.3 (C30), 25.8 (C3), 25.5 (C5'), and 19.7 (C4') ppm.

HRMS m/z 587.5383 $[M + Na]^+$ (calculated for $C_{37}H_{72}O_3Na$, 587.5374).

(9*Z*)-32-((2'-Tetrahydropyranyloxy)dotriacont-9-en-1-yl oleate. To a solution of **4** (293 mg, 0.52 mmol, 1.0 equivalents), EDC·HCl (199 mg, 1.04 mmol, 2.0 equivalents), and 4-dimethylaminopyridine (63 mg, 0.52 mmol, 1.0 equivalents) in dry CH_2Cl_2 (18 mL) and dry pyridine (6 mL) was added oleic acid (175 mg, 0.62 mmol, 1.2 equivalents). The solution was stirred at RT under argon for 23 h, then diluted with CH_2Cl_2 (30 mL) and H_2O (30 mL). The aqueous phase was extracted with CH_2Cl_2 (3 × 30 mL). The combined organic phases were dried over anhydrous Na_2SO_4, filtered, and evaporated under reduced pressure. The crude product was purified by silica gel flash chromatography (*n*-hexane:EtOAc 95:5) and dried *in vacuo* to obtain a white solid (397 mg, 93%, *Z*/*E* ratio > 98:2). R_f = 0.43 (*n*-hexane:EtOAc 9:1).

^{1}H NMR (499.82 MHz, $CDCl_3$, 25 °C): δ_H 5.35 (1H, dtt, $J_{9,11}$ = –1.6, $J_{9,8}$ = 7.2, $J_{9,10}$ = 10.6 Hz, H-9), 5.35 (1H, dtt, $J_{9',11'}$ = –0.7, $J_{9',8'}$ = 7.2, $J_{9',10'}$ = 11.3 Hz, H-9'), 5.34 (1H, dtt, $J_{10,8}$ = –1.4 Hz, $J_{10,11}$ = 6.9 Hz, H-10), 5.34 (1H, dtt, $J_{10',8'}$ = –0.8, $J_{10',11'}$ = 6.9 Hz, H-10'), 4.57 (1H, dd, $J_{2'',3''a}$ = 2.8 Hz, $J_{2'',3''b}$ = 4.6 Hz, H-2''), 4.05 (2H, t, $J_{1,2}$ = 6.7 Hz, H-1), 3.87 (1H, ddd, $J_{6''a,5''a}$ = 2.7, $J_{6''a,5''b}$ = 7.9, $J_{6''a,6''b}$ = −11.4 Hz, H-6''a), 3.73 (1H, dt, $J_{32a,31}$ = 6.9, $J_{32a,32b}$ = −9.6 Hz, H-32a), 3.50 (1H, ddd, $J_{6''b,5''a}$ = 4.1, $J_{6''b,5''b}$ = 5.8 Hz, H-6''b), 3.38 (1H, dt, $J_{32b,31}$ = 6.7 Hz, H-32b), 2.29 (2H, t, $J_{2',3'}$ = 7.5 Hz, H-2'), 2.01 (4H, each ddt, $J_{8,7;11,12}$ = 7.0 Hz, H-8, H-11), 2.01 (4H, each ddt, $J_{8',7'}$ = 7.1, $J_{11',12'}$ = 7.3 Hz, H-8', H-11'), 1.83 (1H, ddddd, $J_{4''a,5''b}$ = 3.1, $J_{4''a,3''b}$ = 3.8, $J_{4''a,5''a}$ = 8.6, $J_{4''a,3''a}$ = 9.2, $J_{4''a,4''b}$ = −12.1 Hz, H-4''a), 1.71 (1H, dddd, $J_{3''a,4''b}$ = 3.7, $J_{3''a,3''b}$ = −12.8 Hz, H-3''a), 1.61 (2H, tt, $J_{2,3}$ = 8.7 Hz, H-2), 1.61 (2H, tt, $J_{3',4'}$ = 7.0 Hz, H-3'), 1.59 (2H, tt, $J_{31,30}$ = 6.7 Hz, H-31), 1.56−1.49 (4H, m, H-3''b, H-4''b, H-5''a, H-5''b), 1.37−1.23 (68H, m, H-3−H-7, H-12−H-30, H-4'−H-7', H-12'−H-17'), and 0.88 (3H, t, $J_{18',17'}$ = 7.3 Hz, H-18') ppm.

^{13}C NMR (125.68 MHz, $CDCl_3$, 25 °C): δ_C 174.0 (C1'), 130.0 (C9, C9'), 129.8 (C10, C10'), 98.9 (C2''), 67.7 (C32), 64.4 (C1), 62.3 (C6''), 34.4 (C2'), 31.9 (C16'), 30.8 (C3''), 29.8 (C31), 29.7−29.1 (C4−C7, C4'−C7', C12−C29, C12'−C15'), 28.7 (C2), 27.2 (C8, C8', C11, C11'), 26.3 (C30), 25.9 (C3), 25.5 (C5''), 25.0 (C3'), 22.7 (C17'), 19.7 (C4''), and 14.1 (C18') ppm.

HRMS m/z 851.7771 $[M + Na]^+$ (calculated for $C_{55}H_{104}O_4Na$, 851.7827).

(9*Z*)-32-Hydroxydotriacont-9-en-1-yl oleate (5). A solution of (9Z)-32-((2'-tetrahydropyranyloxy)dotriacont-9-en-1-yl oleate (390 mg, 0.47 mmol, 1.0 equivalents) and (1S)-(+)-camphor-10-sulfonic acid (16 mg, 0.07 mmol, 0.1 equivalents) in dry CH_3OH (10 mL) and dry THF (7 mL) was stirred at RT under argon for 19 h. The solvents were evaporated under reduced pressure, and the crude product was purified by silica gel flash chromatography (*n*-hexane:EtOAc 4:1) and dried *in vacuo* to obtain a white solid (331 mg, 94%, *Z*/*E* ratio > 98:2). R_f = 0.45 (*n*-hexane:EtOAc 4:1).

^{1}H NMR (499.82 MHz, $CDCl_3$, 25 °C): δ_H 5.35 (1H, dtt, $J_{9,11}$ = −1.6, $J_{9,8}$ = 7.2, $J_{9,10}$ = 10.6 Hz, H-9), 5.35 (1H, ddt, $J_{9',11'}$ = −0.7, $J_{9',8'}$ = 7.2, $J_{9',10'}$ = 11.3 Hz, H-9'), 5.34 (1H, dtt, $J_{10,8}$ = −1.4, $J_{10,11}$ = 6.9 Hz, H-10), 5.34 (1H, dtt, $J_{10',8'}$ = −0.8, $J_{10',11'}$ = 6.9 Hz, H-10'), 4.05 (2H, t, $J_{1,2}$ = 6.7 Hz, H-1), 3.64 (2H, dt, $J_{32,32\text{-}OH}$ = 5.5 Hz, $J_{32,31}$ = 6.6 Hz, H-32), 2.29 (2H, t, $J_{2',3'}$ = 7.6 Hz, H-2'), 2.01 (4H, each ddt, $J_{11,12;8,7}$ = 7.0 Hz, H-8, H-11), 2.01 (4H, each ddt, $J_{8',7'}$ = 7.1, $J_{11',12'}$ = 7.3 Hz, H-8', H-11'), 1.62 (2H, tt, $J_{2,3}$ = 8.0 Hz, H-2), 1.61 (2H, tt, $J_{3',4'}$ = 6.5 Hz, H-3'), 1.57 (2H, tt, $J_{31,30}$ = 7.4 Hz, H-31), 1.37−1.23 (68H, m, H-3−H-7, H-12−H-30, H-4'−H-7', H-12'−H-17'), 1.21 (1H, t, 32-OH),

and 0.88 (3H, t, $J_{18',17'}$ = 7.3 Hz, H-18′) ppm.

^{13}C NMR (125.68 MHz, $CDCl_3$, 25 °C): δ_C 174.0 (C1′), 130.0 (C9′, C9), 129.8 (C10′, C10), 64.4 (C1), 63.1 (C32), 34.4 (C2′), 32.8 (C31), 31.9 (C16′), 29.8−29.1 (C4−C7, C4′−C7′, C12−C29, C12′−C15′), 28.7 (C2), 27.2 (C8, C11, C8′, C11′), 25.9 (C3), 25.8 (C30), 25.0 (C3′), 22.7 (C17′), and 14.1 (C18′) ppm.

HRMS m/z 745.7427 $[M + H]^+$ (calculated for $C_{50}H_{97}O_3$, 745.7432).

(23*Z*)-32-Oleoyloxydotriacont-23-enoic acid (6). To a solution of **5** (222 mg, 0.30 mmol, 1.0 equivalents) in THF (4 mL), acetone (8 mL), and EtOAc (4 mL) was added Jones reagent (CrO_3/H_2SO_4 2.0 mol/L, 447 µL, 0.89 mmol, 3.0 equivalents), and the resulting brown mixture was stirred vigorously at RT for 1.5 h. The reaction mixture was quenched with 2-propanol (5 mL) and filtered through diatomaceous earth. The diatomaceous earth was washed with Et_2O (100 mL), and the filtrate was washed with acidified brine (40 mL brine + 10 mL 1 M HCl). The combined organic phases were dried over anhydrous Na_2SO_4, filtered, and evaporated under reduced pressure. The crude product was purified by silica gel flash chromatography (*n*-hexane:EtOAc:AcOH 9:1:0.01 → 7:3:0.01) and dried *in vacuo* to obtain a white solid (204 mg, 90%, *Z*/*E* ratio > 98:2). Mp = 59.9 ± 0.3 °C; R_f = 0.52 (*n*-hexane:EtOAc:AcOH 7:3:0.01).

^{1}H NMR (499.82 MHz, $CDCl_3$, 25 °C): δ_H 10.21 (1H, br s, 2-COOH), 5.35 (1H, dtt, $J_{9',11'}$ = –0.8, $J_{9',8'}$ = 7.2, $J_{9',10'}$ = 11.6 Hz, H-9′), 5.35 (1H, dtt, $J_{24,22}$ = –1.3, $J_{24,25}$ = 7.2, $J_{24,23}$ = 11.5 Hz, H-24), 5.34 (1H, dtt, $J_{10',8'}$ = –1.0, $J_{10',11'}$ = 6.9 Hz, H-10′), 5.34 (1H, dtt, $J_{23,25}$ = –1.0, $J_{23,22}$ = 6.9 Hz, H-23), 4.05 (2H, t, $J_{32,31}$ = 6.7 Hz, H-32), 2.35 (2H, t, $J_{2,3}$ = 7.5 Hz, H-2), 2.29 (2H, t, $J_{2',3'}$ = 7.6 Hz, H-2′), 2.01 (4H, each ddt, $J_{8',7'}$ = 7.1, $J_{11',12'}$ = 7.3 Hz, H-8′, H-11′), 2.01 (4H, each ddt, $J_{22,21;25,26}$ = 7.2 Hz, H-22, H-25), 1.63 (2H, tt, $J_{3,4}$ = 7.2 Hz, H-3), 1.61 (2H, tt, $J_{3',4'}$ = 7.2 Hz, H-3′), 1.61 (2H, tt, $J_{31,30}$ = 7.3 Hz, H-31), 1.37−1.23 (66H, m, H-4−H-21, H-4′−H-7′, H-12′−H-17′, H-26−H-30), and 0.88 (3H, t, $J_{18',17'}$ = 7.2 Hz, H-18′) ppm.

^{13}C NMR (125.68 MHz, $CDCl_3$, 25 °C): δ_C 178.3 (C1), 174.0 (C1′), 130.0 (C9′, C24), 129.8 (C10′, C23), 64.4 (C32), 34.4 (C2′), 33.8 (C2), 31.9 (C16′), 29.8−29.1 (C4−C21, C4′−C7′, C12′−C15′, C26−C29), 28.7 (C31), 27.2 (C8′, C11′, C22, C25), 25.9 (C30), 25.0 (C3′), 24.7 (C3), 22.7 (C17′), and 14.1 (C18′) ppm.

HRMS m/z 757.7084 $[M - H]^-$ (calculated for $C_{50}H_{93}O_4$, 757.7079).

(9*Z*)-1,32-Dioleoyloxydotriacont-9-ene (7). To a solution of **5** (101 mg, 0.14 mmol, 1.0 equivalents), EDC·HCl (52 mg, 0.27 mmol, 2.0 equivalents), and 4-dimethylaminopyridine (17 mg, 0.14 mmol, 1.0 equivalents) in dry CH_2Cl_2 (4.0 mL) and dry pyridine (1.5 mL) was added oleic acid (50 mg, 0.18 mmol, 1.3 equivalents). The solution was stirred at RT under argon for 45 h, then diluted with CH_2Cl_2 (10 mL). The organic phase was washed with 1 M HCl (2 × 10 mL) and saturated aqueous $NaHCO_3$ (20 mL). The separate aqueous phases were washed with CH_2Cl_2 (4 × 10 mL). The combined organic phases were dried over anhydrous Na_2SO_4, filtered, and evaporated under reduced pressure. The crude product was purified by silica gel flash chromatography (*n*-hexane:EtOAc 95:5) and dried *in vacuo* to obtain a white solid (133 mg, 97%, *Z*/*E* ratio > 98:2). Mp = 37.5 ± 0.2 °C; R_f = 0.47 (*n*-hexane:EtOAc 9:1).

^{1}H NMR (499.82 MHz, $CDCl_3$, 25 °C): δ_H 5.35−5.34 (6H, m, H-9, H-10, H-9′, H-10′, H-9′′, H-10′′), 4.05 (4H, each t, $J_{1,2;32,31}$ = 6.8 Hz, H-1, H-32), 2.29 (4H, each t, $J_{2',3';2'',3''}$ = 7.5 Hz, H-2′, H-2′′), 2.01 (4H, each ddt, $J_{8,10}$ = −2.0, $J_{11,9}$ = −2.4, $J_{8,7;11,12}$ = 7.0, $J_{11,10}$ = 7.7, $J_{8,9}$ = 7.8 Hz, H-8, H-11), 2.01 (8H, each ddt, $J_{8',10';8'',10''}$ = −0.6, $J_{11',9';11'',9''}$ = −0.8, $J_{8',9';8'',9''}$ = 6.6, $J_{11',10';11''10''}$ = 6.7, $J_{8',7';8'',7''}$ = 7.1, $J_{11',12';11'',12''}$ = 7.3 Hz, H-8′, H-8′′, H-11′, H-11′′), 1.62 (2H, tt, $J_{2,3}$ = 8.0 Hz, H-2), 1.61 (4H, each tt, $J_{3',4';3'',4''}$ = 6.2 Hz, H-3′, H-3′′), 1.61 (2H, tt, $J_{31,30}$ = 7.4 Hz, H-31) 1.37−1.23 (88H, m, H-3−H-7, H-4′−H-7′, H-12−H-30, H-4′′−H-7′′, H-12′−H17′, H-12′′−H-17′′), and 0.88 (6H, each t, $J_{18',17';18'',17''}$ = 7.2 Hz, H-18′, H-18′′) ppm.

^{13}C NMR (125.68 MHz, $CDCl_3$, 25 °C): δ_C 174.0 (C1′, C1′′), 130.0 (C9, C9′, C9′′), 129.8 (C10, C10′, C10′′), 64.4 (C1, C32), 34.4 (C2′, C2′′), 31.9 (C16′, C16′′), 29.8−29.1 (C4−C7, C4′−C7′, C4′′−C7′′, C12−C29, C12′−C15′, C12′′−C15′′), 28.7 (C2, C31), 27.2 (C8, C8′, C8′′, C11, C11′, C11′′), 26.0 (C3, C30), 25.0 (C3′, C3′′), 22.7 (C17′, C17′′), and 14.1 (C18′, C18′′) ppm.

HRMS m/z 1031.9705 $[M + Na]^+$ (calculated for $C_{68}H_{128}O_4Na$, 1031.9705).

3. NMR spectra of synthesized compounds

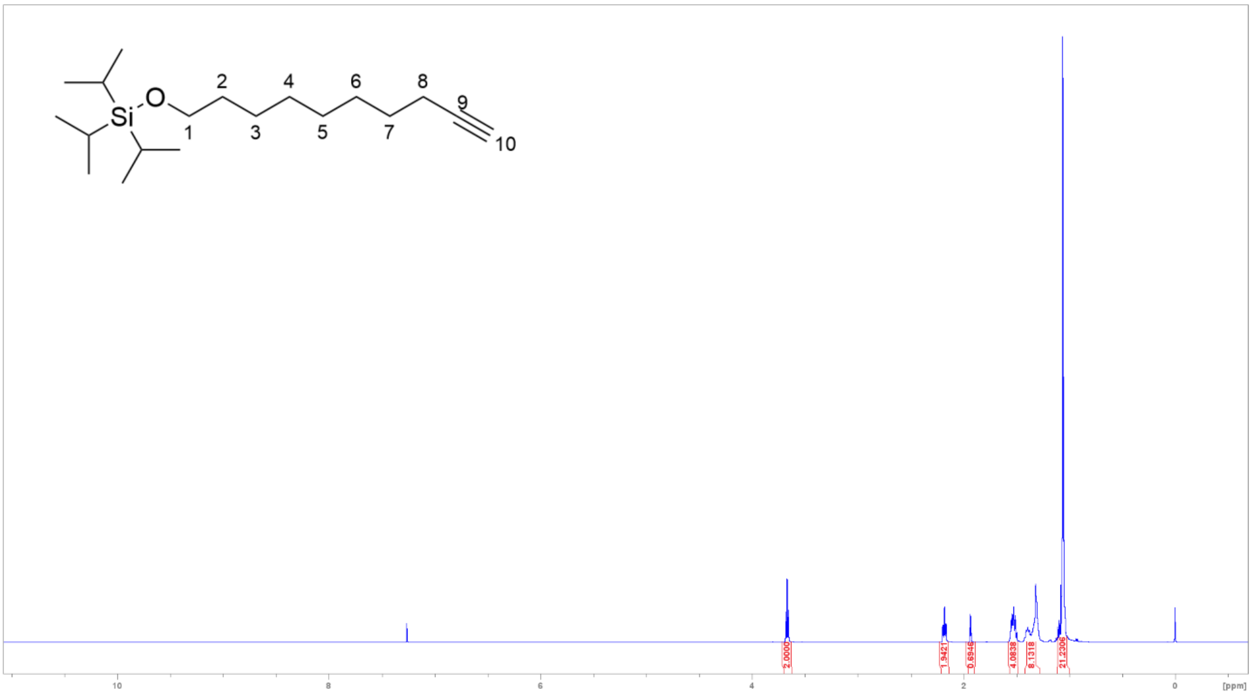

Figure S6. ^{1}H NMR (499.82 MHz) of **1** at 25°C.

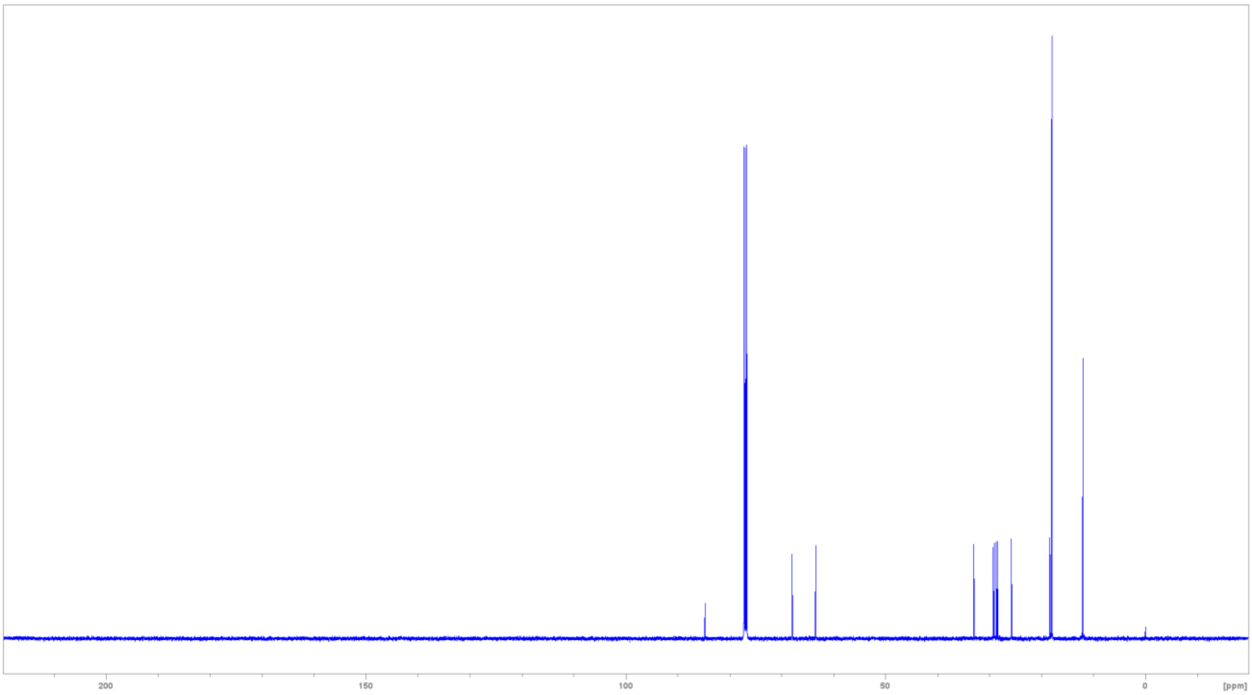

Figure S7. ^{13}C NMR (125.68 MHz) of **1** at 25°C.

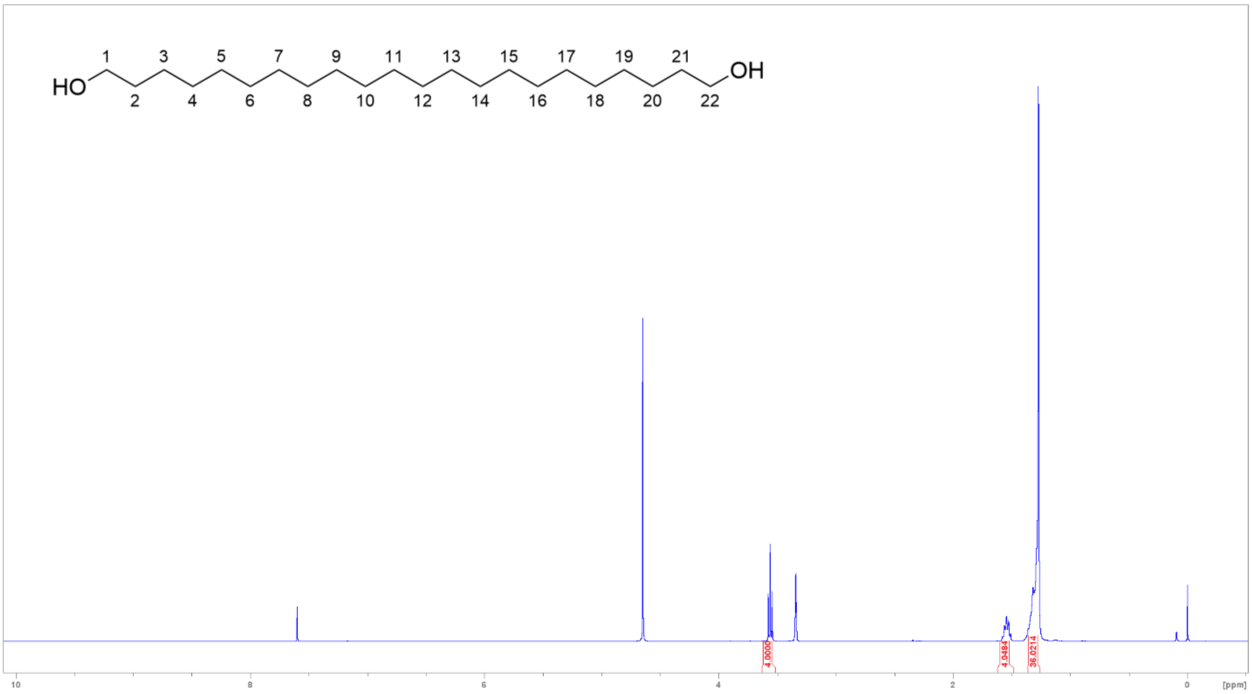

Figure S8. ^{1}H NMR (499.82 MHz) of 1,22-docosanediol at 25°C.

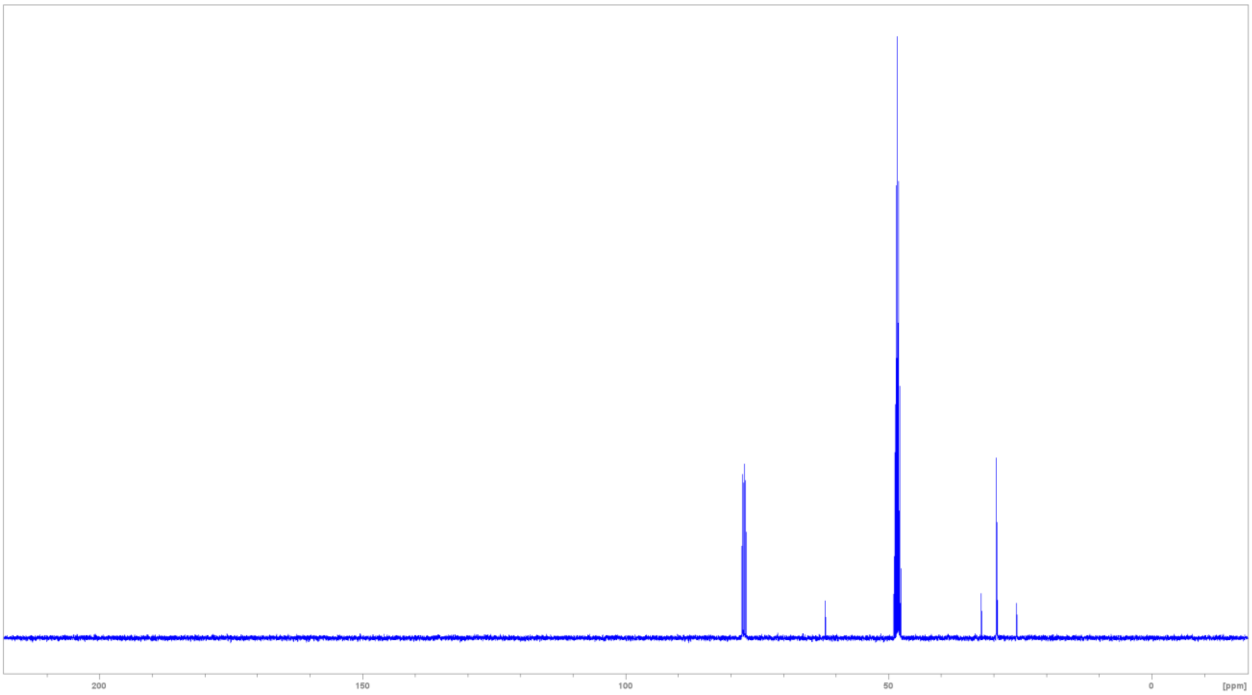

Figure S9. ^{13}C NMR (125.68 MHz) of 1,22-docosanediol at 25°C.

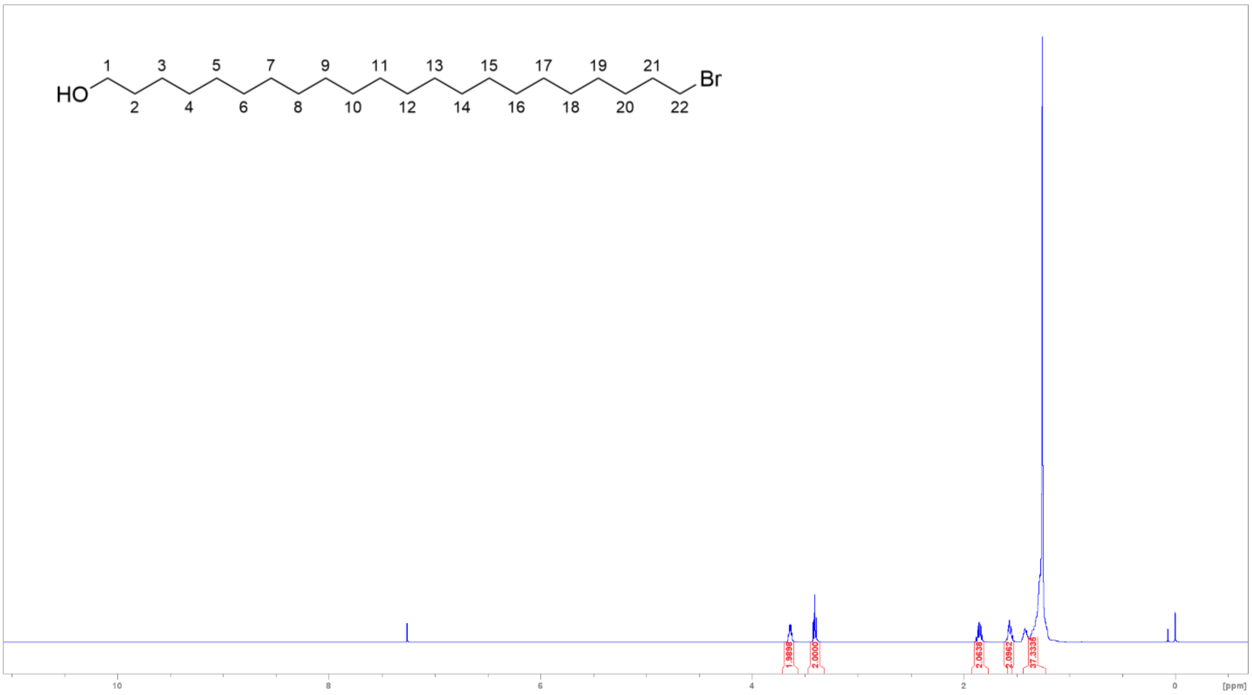

Figure S10. ^{1}H NMR (499.82 MHz) of 22-bromo-1-docosanol at 25°C.

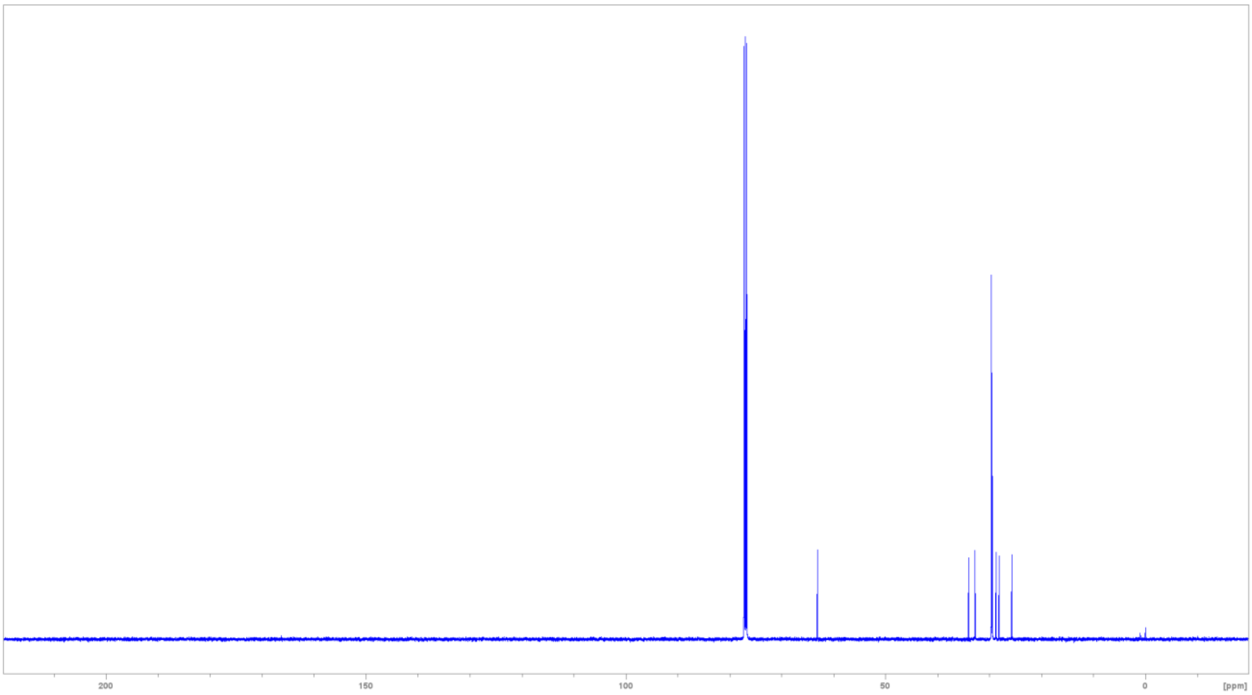

Figure S11. ^{13}C NMR (125.68 MHz) of 22-bromo-1-docosanol diol at 25°C.

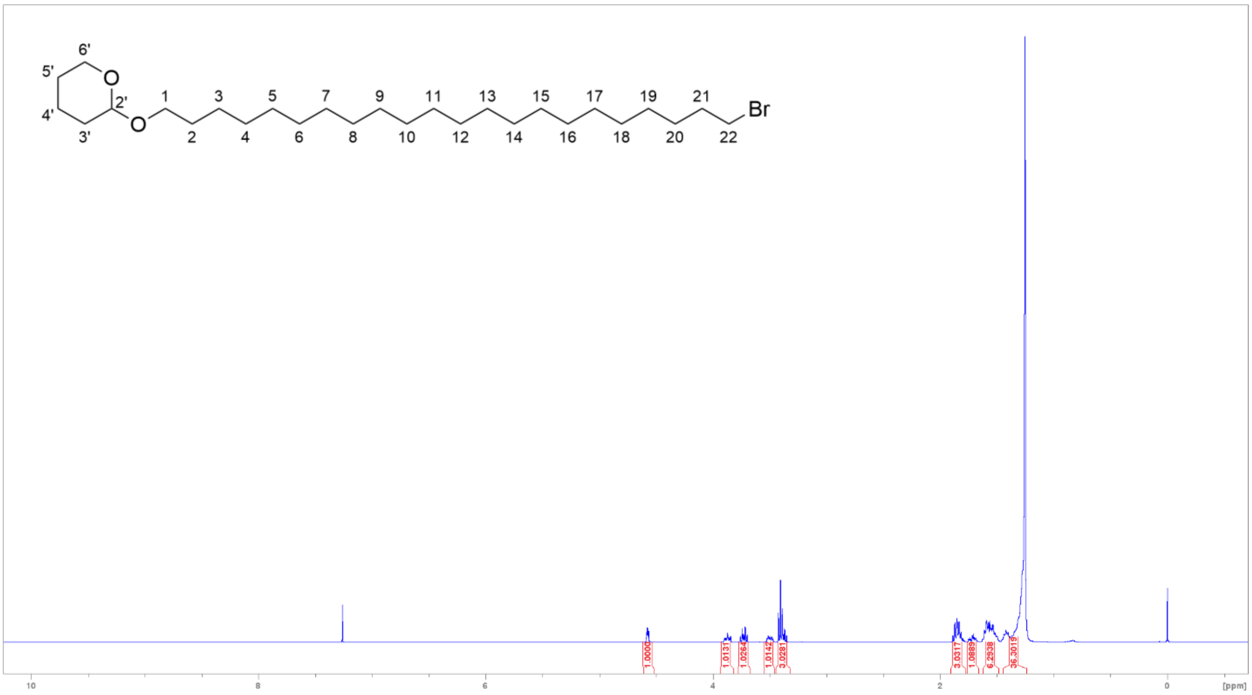

Figure S12. ^{1}H NMR (499.82 MHz) of **2** at 25°C.

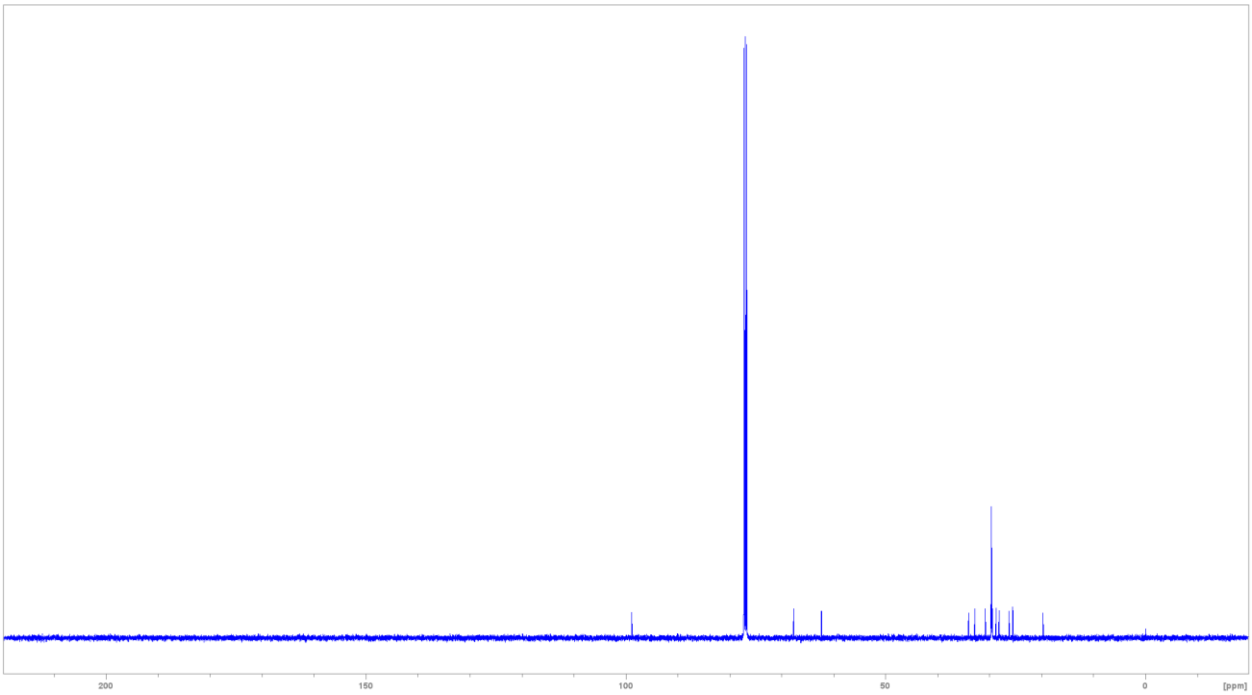

Figure S13. ^{13}C NMR (125.68 MHz) of **2** at 25°C.

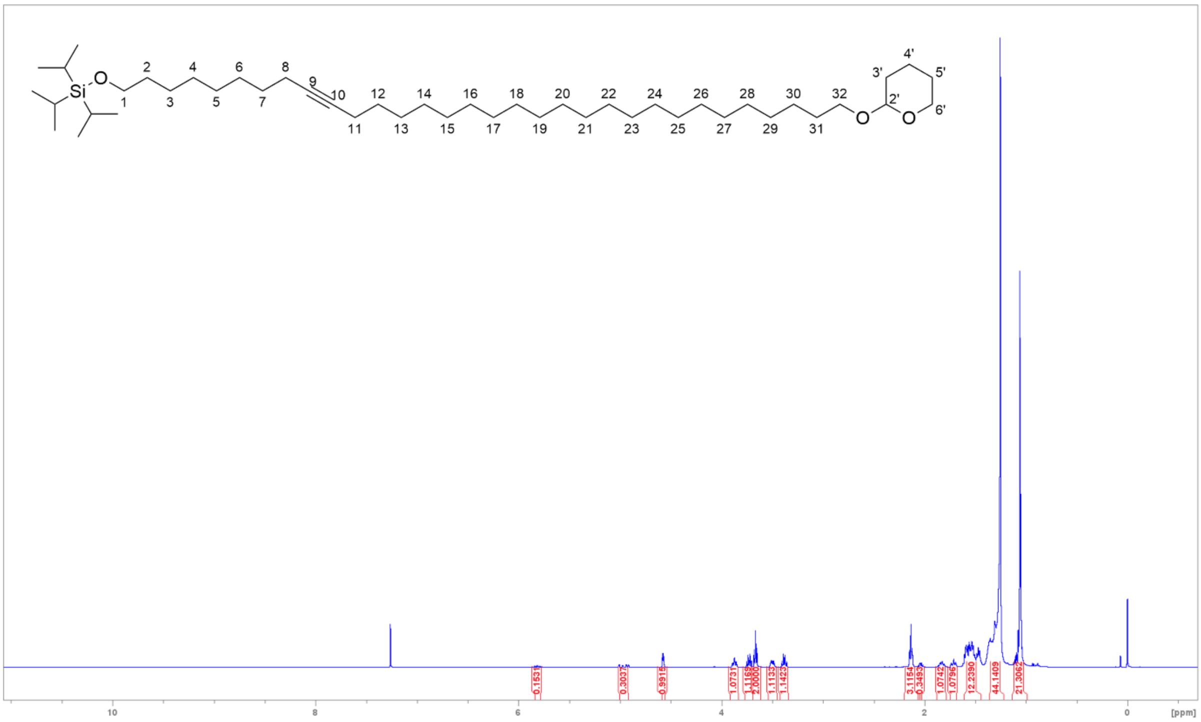

Figure S14. ^{1}H NMR (499.82 MHz) of **3** at 25°C.

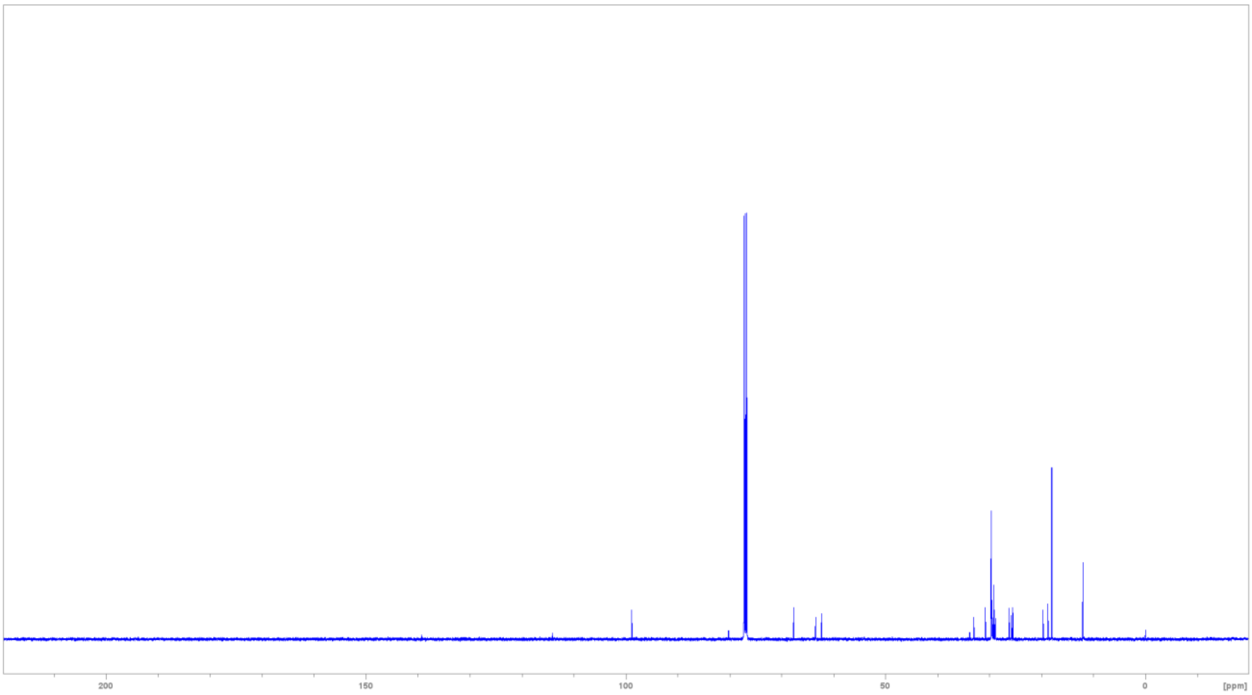

Figure S15. ^{13}C NMR (125.68 MHz) of **3** at 25°C.

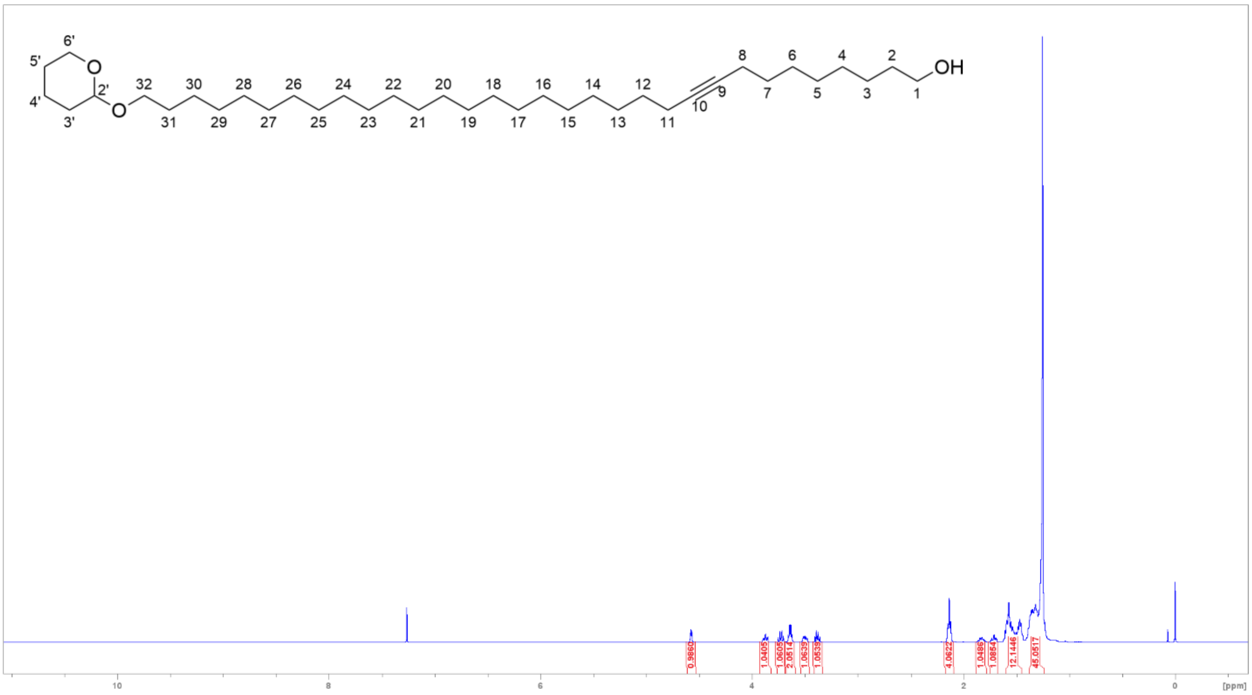

Figure 16. ^{1}H NMR (499.82 MHz) of 32-(2'-tetrahydropyranyloxy)dotriacont-9-yn-1-ol at 25°C.

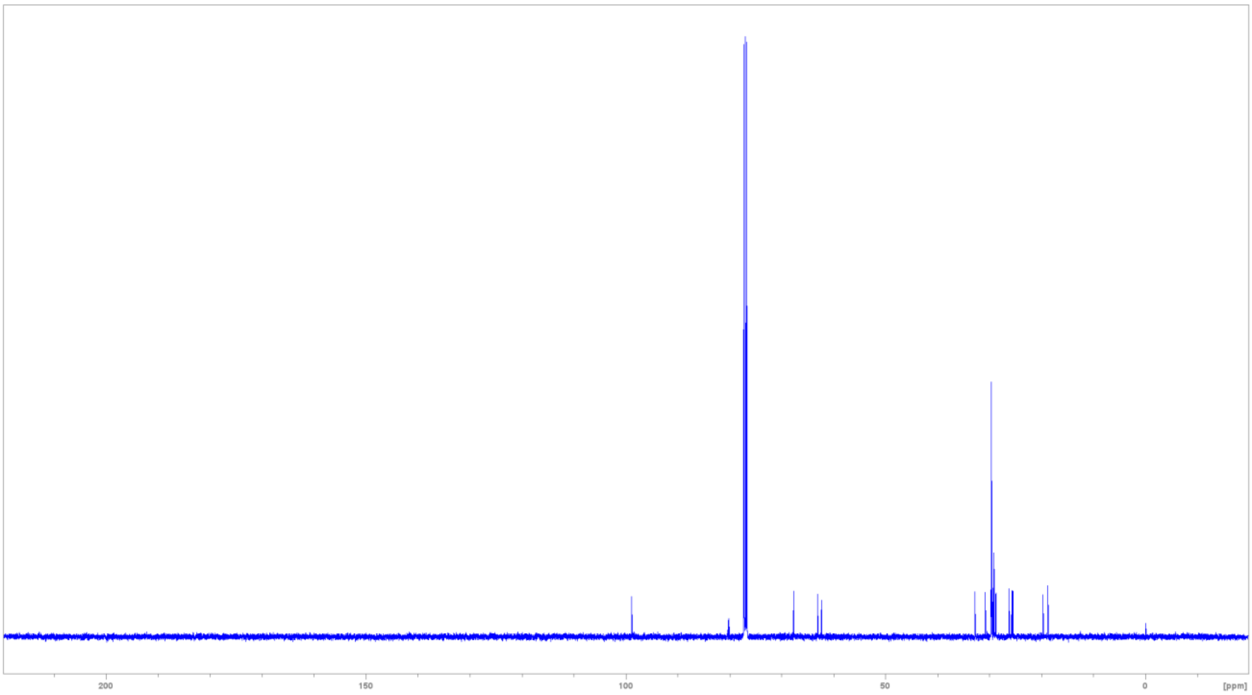

Figure S17. ^{13}C NMR (125.68 MHz) of 32-(2'-tetrahydropyranyloxy)dotriacont-9-yn-1-ol at 25°C.

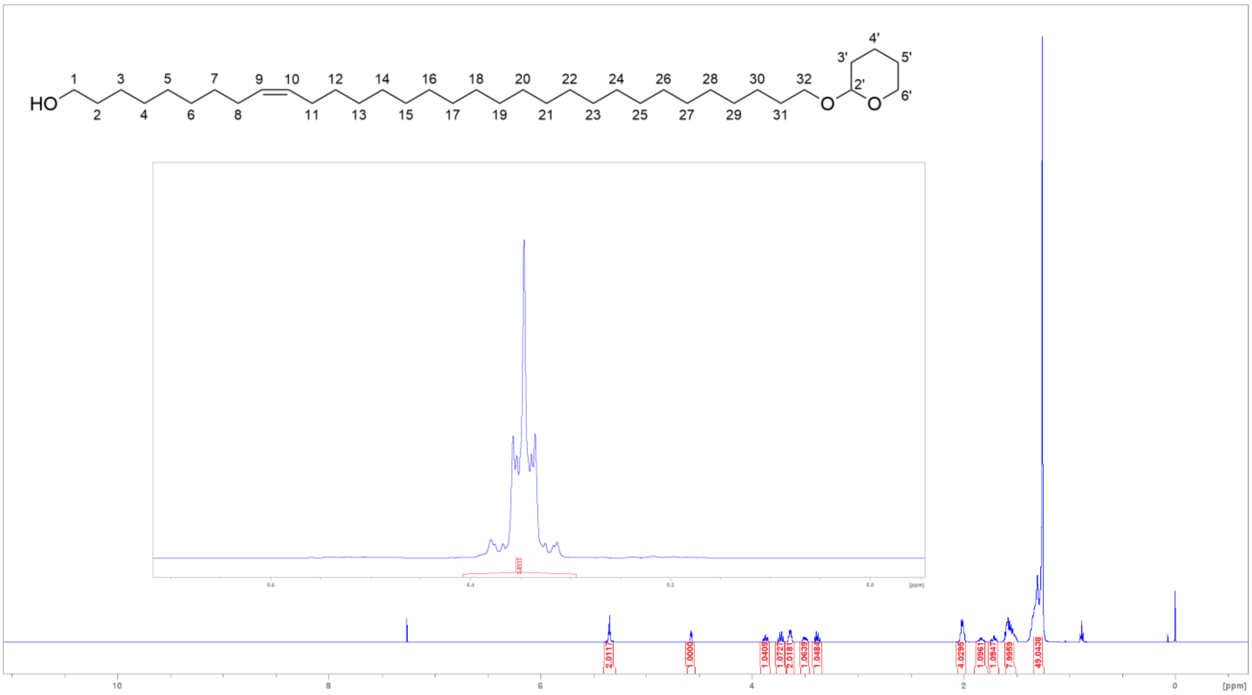

Figure S18. ^{1}H NMR (499.82 MHz) of **4** at 25°C with double bond signals highlighted.

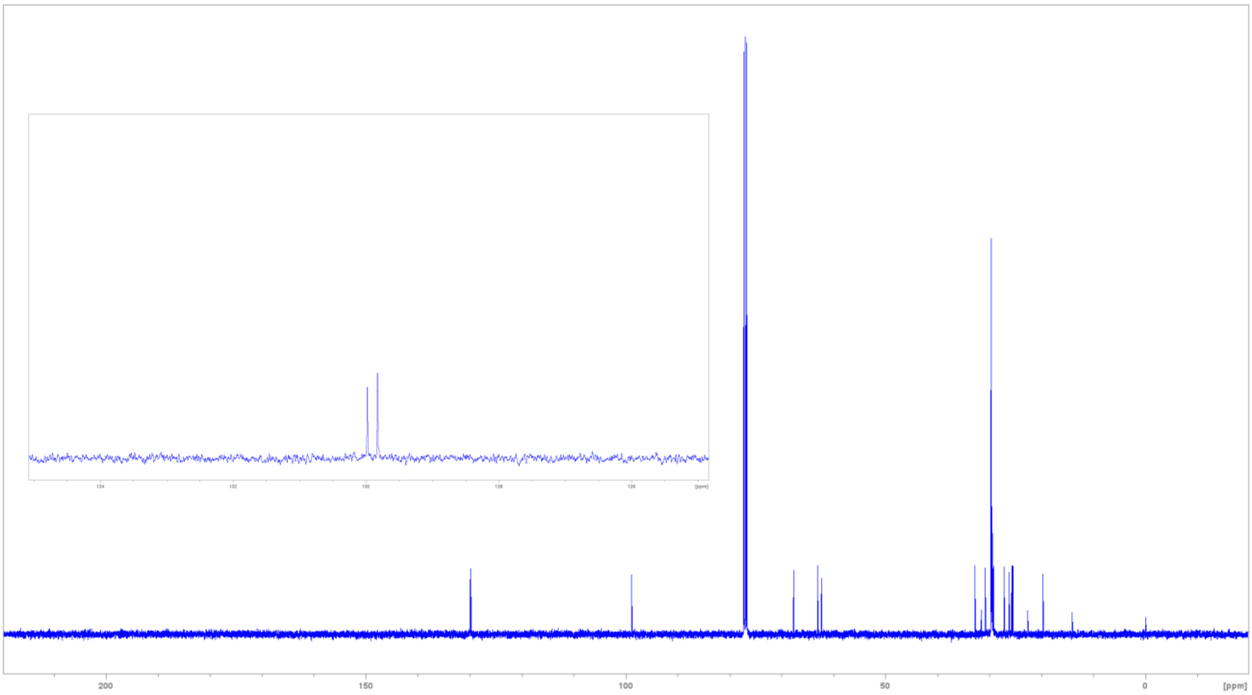

Figure S19. ^{13}C NMR (125.68 MHz) of **4** at 25°C with double bond signals highlighted.

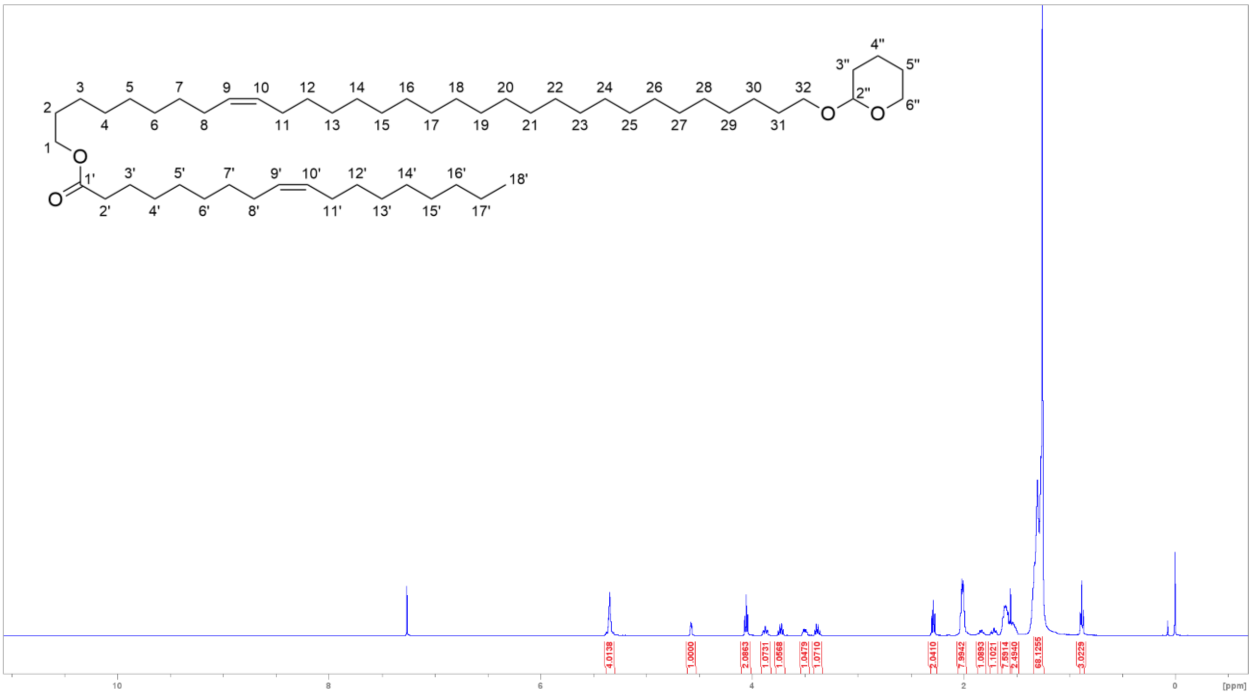

Figure S20. ^{1}H NMR (499.82 MHz) of (9*Z*)-1-oleoyloxy-32-(2’-tetrahydropyranyloxy)dotriacont-9-ene at 25°C.

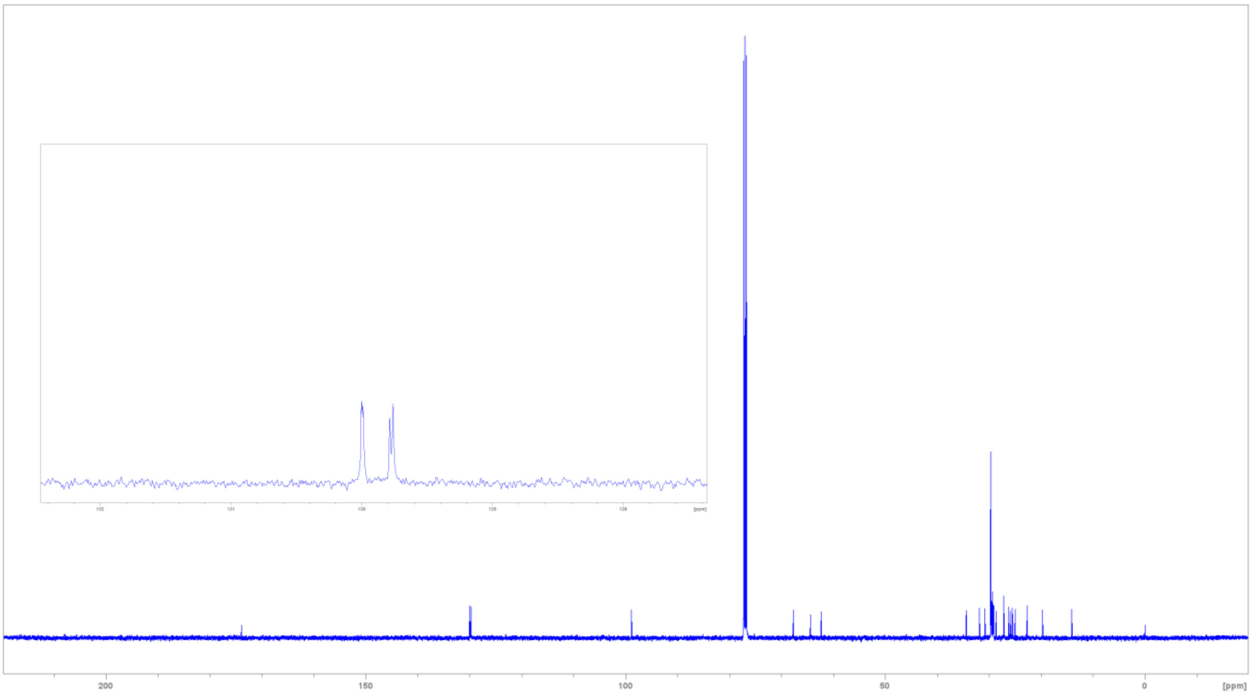

Figure S21. ^{13}C NMR (125.68 MHz) of (9*Z*)-1-oleoyloxy-32-(2’-tetrahydropyranyloxy)dotriacont-9-ene at 25°C with double bond signals highlighted.

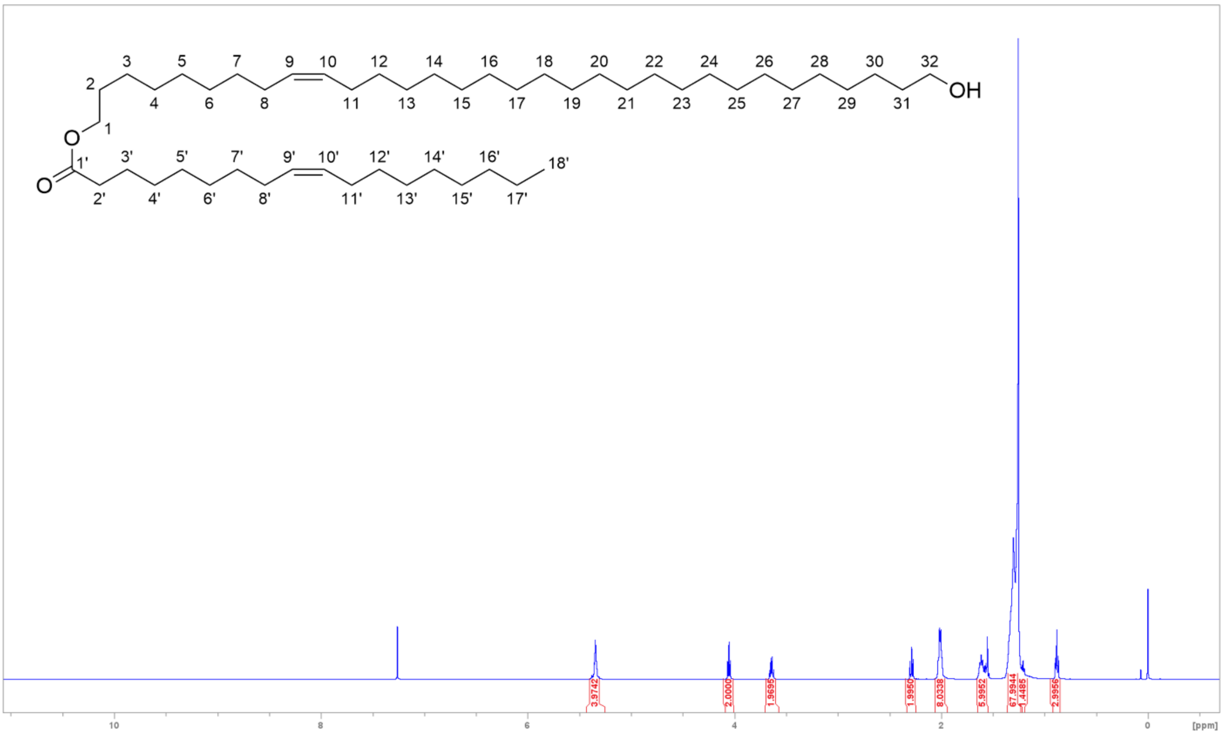

Figure S22. ^{1}H NMR (499.82 MHz) of **5** at 25°C.

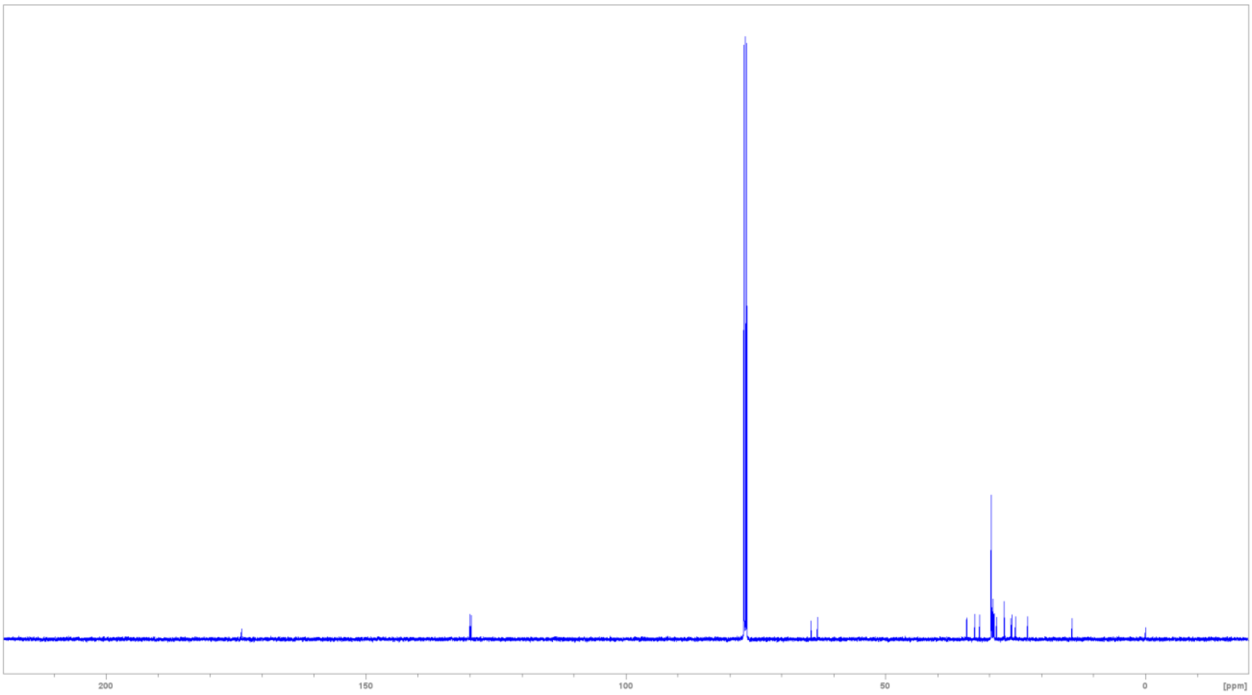

Figure S23. ^{13}C NMR (125.68 MHz) of **5** at 25°C.

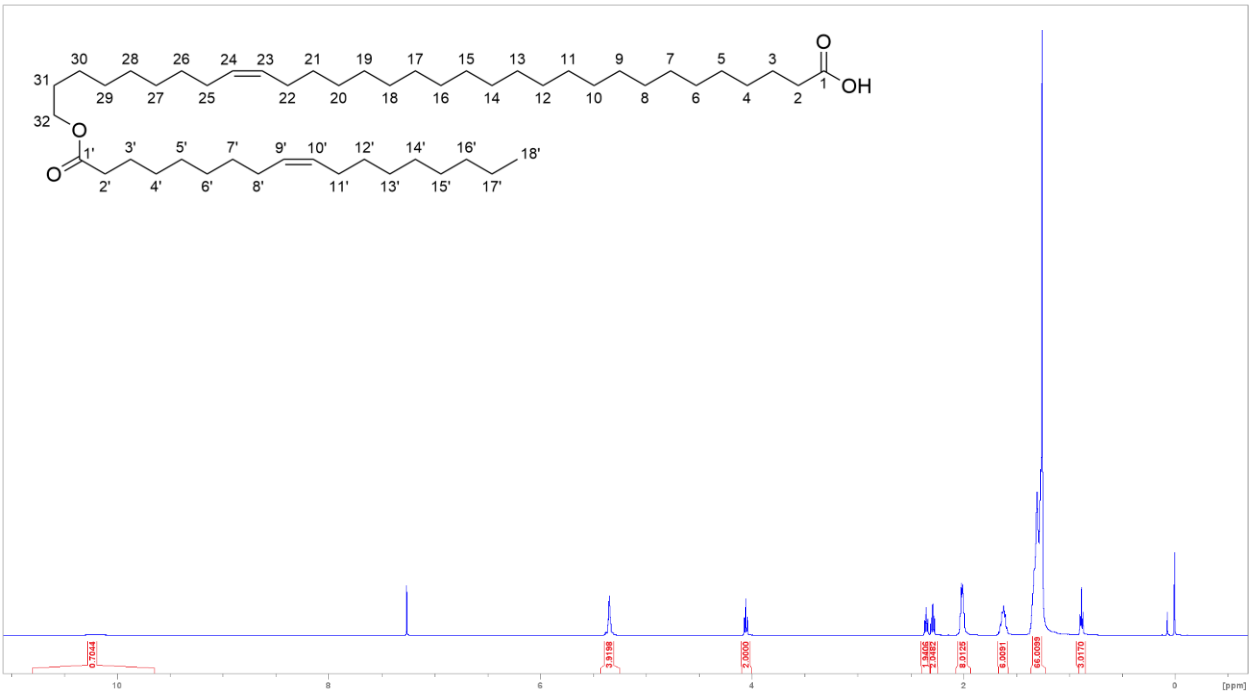

Figure S24. ^{1}H NMR (499.82 MHz) of **6** (32:1/18:1-OAHFA) at 25°C.

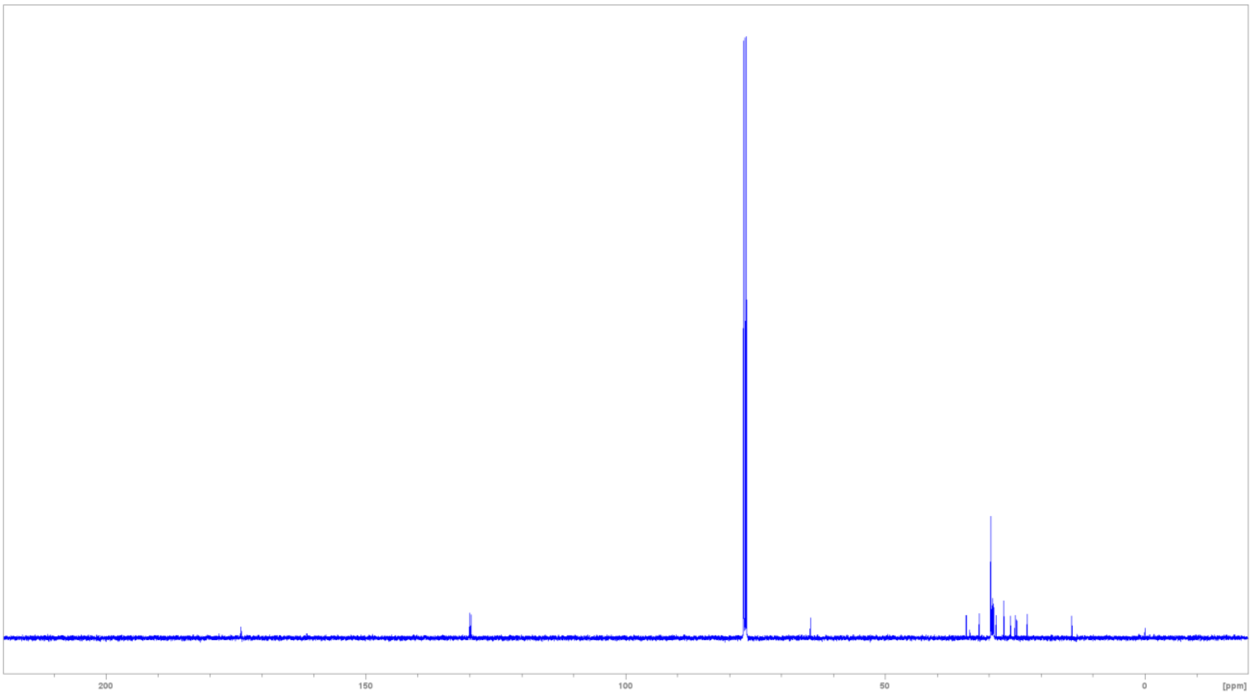

Figure S25. ^{13}C NMR (125.68 MHz) of **6** (32:1/18:1-OAHFA) at 25°C.

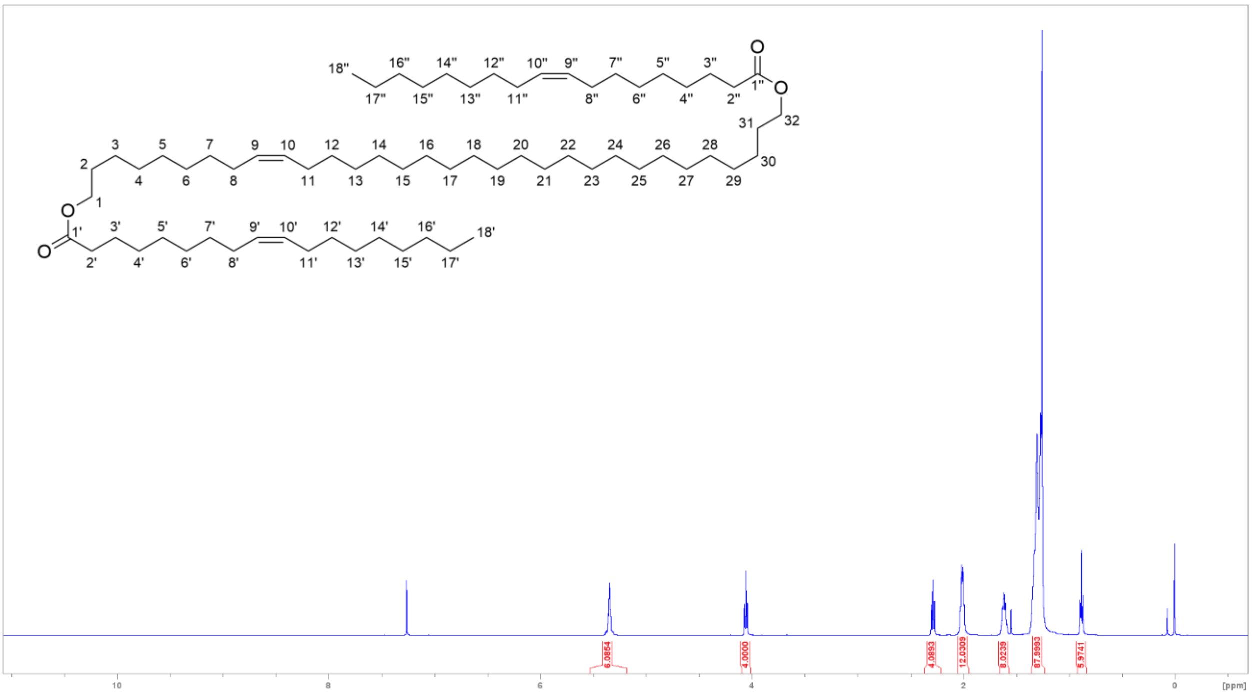

Figure S26. ^{1}H NMR (499.82 MHz) of **7** (18:1/32:1/18:1-DiE) at 25°C.

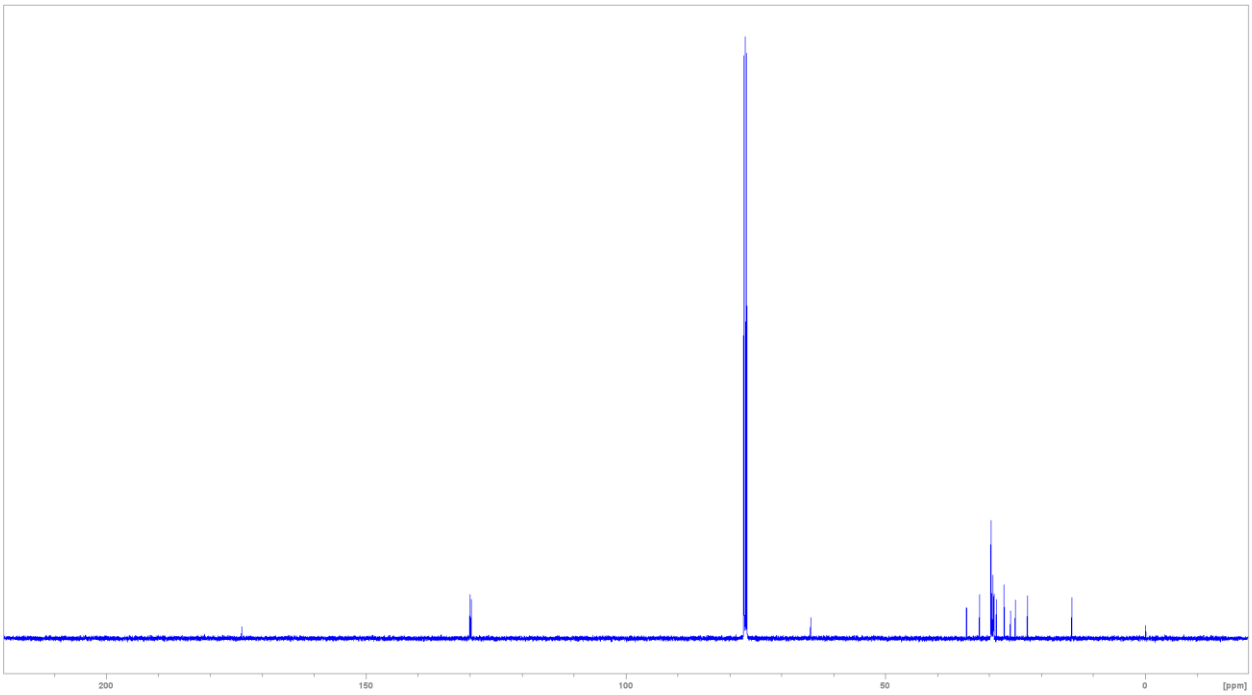

Figure S27. ^{13}C NMR (125.68 MHz) of **6** (18:1/32:1/18:1-DiE) at 25°C.

4. Supplementary Langmuir trough experiments

The supplementary Langmuir trough experiments consisted of measuring surface pressure isotherms of the films formed by 32:1/18:1-OAHFA and 18:1/32:1/18:1-type II DiE at ocular surface temperature and imaging the film structure at selected surface pressures utilizing Brewster angle microscopy (BAM). Below is a short description of the instrumentation utilized and the results are displayed in **Figure S28**.

These Langmuir trough experiments were conducted with a KSV NIMA Langmuir large trough (Biolin Scientific, Espoo, Finland; dimensions 580 × 145 mm) equipped with a temperature sensor, a surface pressure balance with a platinum Wilhelmy plate, and a Brewster angle microscope (KSV NIMA microBAM, Espoo, Finland). The temperature was controlled by a circulating water bath (LAUDA ECO E4, Germany) and maintained at 35 ± 1 °C during the measurements. To prevent oxidation of the samples by ambient ozone, the device was contained within an acrylic enclosure (volume 290 L) and dry air was continuously passed through an ODS-3 P ozone destruct unit (Hull, Iowa) into the enclosure at a rate of 76 L/min.

The Langmuir trough was initially filled with a PBS buffer (140 mM NaCl, 3 mM KCl, 10 mM phosphate buffer, pH 7.4) which acted as the aqueous sub-phase during the measurements. The tear film lipids were spread from 5 mM chloroform solutions onto the aqueous sub-phase and the chloroform was allowed to evaporate for 5 min before starting the measurements. The films were compressed at a rate of 10 mm/min.

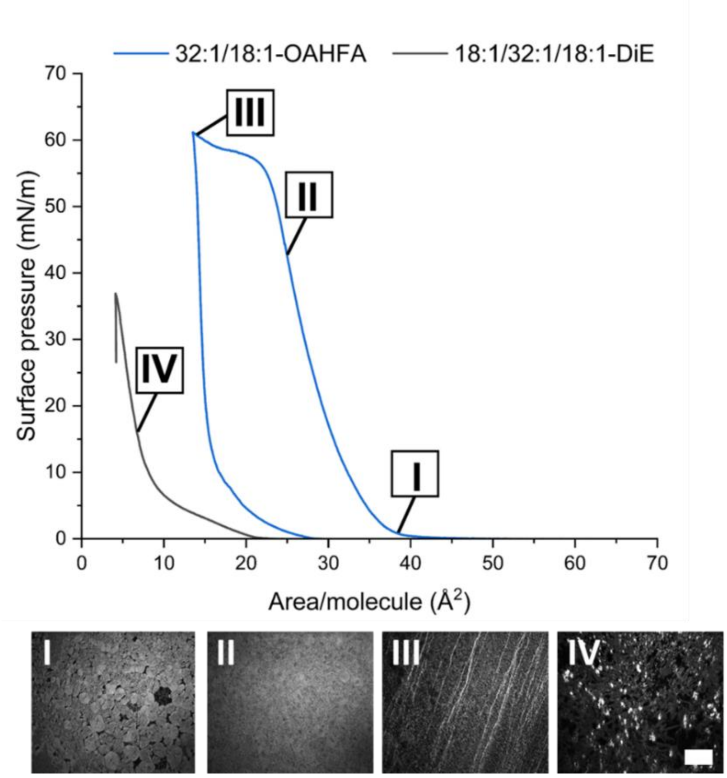

Figure S28. Top: Surface pressure isotherms of 32:1/18:1-OAHFA and 18:1/32:1/18:1-DiE are displayed as a function of area/molecule (Å^2). **Bottom:** Representative BAM images are shown highlighting the appearance of the films formed. The scale bar depicts 500 µm.

5. References

(1) Stubb, H.; Viitaja, T.; Trevorah, R. M.; Raitanen, J. E.; Moilanen, J.; Svedström, K. J.; Ekholm, F. S. Another Brick in the Wall of Tear Film Insights Added Through the Total Synthesis and Biophysical Profiling of anteiso-Branched Wax and Cholesteryl Esters. *J. Nat. Prod.* **2024**, *87* (4), 954-965. DOI: 10.1021/acs.jnatprod.3c01247.

(2) Tiainen, M.; Soininen, P.; Laatikainen, R. Quantitative Quantum Mechanical Spectral Analysis (qQMSA) of 1H NMR Spectra of Complex Mixtures and Biofluids. *J. Magnetic Res.* **2014**, *242*, 67–78. DOI: 10.1016/j.jmr.2014.02.008.

(3) Trevorah, R. M.; Viljanen, M.; Viitaja, T.; Stubb, H.; Sevon, J.; Konovalov, O.; Jankowski, M.; Fontaine, P.; Hemmerle, A.; Raitanen, J. E.; Ekholm, F. S.; Svedström, K. J. New Insights into the Molecular Structure of Tear Film Lipids Revealed by Surface X-ray Scattering. *J. Phys. Chem. Lett.* **2024**, 15, 316. DOI: 10.1021/acs.jpclett.3c02958.

(4) Viitaja, T.; Raitanen, J. E.; Moilanen, J.; Paananen, R. O.; Ekholm, F. S. Biophysical profiling of synthetic ultra-long tear film lipids. *Colloids Surf. B Biointerfaces* **2023**, 223, 113145. DOI: 10.1016/j.colsurfb.2023.113145.

(5) Viitaja, T.; Raitanen, J. E.; Hynynen, A.; Moilanen, J.; Svedstrom, K.; Paananen, R. O.; Ekholm, F. S. On the importance of chain branching in tear film lipid layer wax and cholesteryl esters. *Colloids Surf B Biointerfaces* **2022**, 214, 112429. DOI: 10.1016/j.colsurfb.2022.112429.
